# Better Agreement of Human Transcriptomic and Proteomic Cancer Expression Data at the Molecular Pathway Activation Level

**DOI:** 10.3390/ijms23052611

**Published:** 2022-02-26

**Authors:** Mikhail Raevskiy, Maxim Sorokin, Galina Zakharova, Victor Tkachev, Nicolas Borisov, Denis Kuzmin, Kristina Kremenchutckaya, Alexander Gudkov, Dmitry Kamashev, Anton Buzdin

**Affiliations:** 1 I.M. Sechenov First Moscow State Medical University, 119991 Moscow, Russia; raevskymichail@gmail.com; 2 OmicsWay Corp., Walnut, CA 91789, USA; sorokin@oncobox.com (M.S.); galina.s.zakharova@gmail.com (G.Z.); dr.gudkov@gmail.com (A.G.); dkamashev@gmail.com (D.K.); 3 Shemyakin-Ovchinnikov Institute of Bioorganic Chemistry, 117997 Moscow, Russia; 4 Oncobox Ltd., 121205 Moscow, Russia; tkachev@oncobox.com; 5 Moscow Institute of Physics and Technology, 141701 Dolgoprudny, Russia; borisov@oncobox.com (N.B.); kuzmin.dv@mipt.ru (D.K.); krem-kristina@mail.ru (K.K.)

**Keywords:** transcriptomics, proteomics, gene expression, intracellular molecular pathways, pathway activation level, human cancer tissue

## Abstract

Previously, we have shown that the aggregation of RNA-level gene expression profiles into quantitative molecular pathway activation metrics results in lesser batch effects and better agreement between different experimental platforms. Here, we investigate whether pathway level of data analysis provides any advantage when comparing transcriptomic and proteomic data. We compare the paired proteomic and transcriptomic gene expression and pathway activation profiles obtained for the same human cancer biosamples in The Cancer Genome Atlas (TCGA) and the NCI Clinical Proteomic Tumor Analysis Consortium (CPTAC) projects, for a total of 755 samples of glioblastoma, breast, liver, lung, ovarian, pancreatic, and uterine cancers. In a CPTAC assay, expression levels of 15,112 protein-coding genes were profiled using the Thermo QE series of mass spectrometers. In TCGA, RNA expression levels of the same genes were obtained using the Illumina HiSeq 4000 engine for the same biosamples. At the gene level, absolute gene expression values are compared, whereas pathway-grade comparisons are made between the pathway activation levels (PALs) calculated using average sample-normalized transcriptomic and proteomic profiles. We observed remarkably different average correlations between the primary RNA- and protein expression data for different cancer types: Spearman Rho between 0.017 (*p* = 1.7 × 10^−13^) and 0.27 (*p* < 2.2 × 10^−16^). However, at the pathway level we detected overall statistically significantly higher correlations: averaged Rho between 0.022 (*p* < 2.2 × 10^−16^) and 0.56 (*p* < 2.2 × 10^−16^). Thus, we conclude that data analysis at the PAL-level yields results of a greater similarity when comparing high-throughput RNA and protein expression profiles.

## 1. Introduction

Most known human gene products execute their molecular function at the protein level. Proteomics, therefore, should theoretically be considered the preferred approach for high-throughput screening of the expression of such genes. This is also true for quantitative assessments of the activities of molecular pathways. However, for various reasons, the current limitations of proteomic techniques do not allow routine screening of most protein-coding genes [1]. For example, in the US National Cancer Institute’s large-scale project *Clinical Proteomic Tumor Analysis Consortium* (CPTAC), devoted to the integrative proteogenomic characterization of human cancers, expression levels for only 15,112 genes have been resolved at the protein level by using tandem mass (MS/MS) spectrometry conducted on Thermo QE series of mass spectrometers (QE, QEplus, QE-HF, and QE-HF-X) [2]. These limitations are especially severe for the analysis of formalin-fixed paraffin-embedded (FFPE) cancer tissue samples, which is a routine format of biosample storage in clinical oncology [3]. Formalin fixation produces numerous artifact chemical modifications, including covalent cross-links between unrelated protein molecules [4].

Thus, it is important to improve current proteomic instruments or to use alternative methods that could be validated at the proteomic level. Transcriptomics offer another way of measuring expression of protein-coding genes [5]. It has been shown that RNA- and proteomic raw gene expression levels statistically significantly correlate with mean average Speaman Rho 0.16–0.52 [6,7,8]. Furthermore, RNA sequencing (RNAseq) can be used as an alternative to immunohistochemical methods for the assessment of biomarker functional status in clinical cancer samples for proteins such as HER2, ESR1, PGR, and PD-L1 [9].

The RNAseq results demonstrated high reproducibility and robustness in resolving transcripts of 23,248 protein-coding genes [10]. This allowed us to incorporate this type of analysis into the pipeline of tumor molecular analysis to estimate the expression of cancer drug target genes and the activation of targeted molecular pathways [11]. The latter, in turn, made it possible to simulate and predict the activities of cancer drugs with known molecular specificities, and to determine their patient-specific rating [12]. However, to our knowledge, the question of whether there is a correlation between the quantitative molecular pathway activation data received using RNAseq and proteomic data has never been explored before.

We have shown previously that the aggregation of RNA-level gene expression profiles into quantitative molecular pathway activation metrics results in reduced batch effects and better agreement between different experimental platforms. However, to our knowledge, the question of whether the pathway level of data analysis provides any advantage when comparing transcriptomic and proteomic data has never been explored before.

Here, we examined this question using paired proteomic and transcriptomic gene expression profiles obtained for the same human cancer tissue biosamples and available through The Cancer Genome Atlas (TCGA) and the NCI Clinical Proteomic Tumor Analysis Consortium (CPTAC) portals. We analyzed a total of 755 samples for glioblastoma and breast, liver, lung, ovarian, pancreatic, and uterine cancers. In CPTAC proteomic assay, the expression levels of 15,112 genes were profiled using the tandem mass (MS/MS) spectrometry conducted on Thermo QE series of mass spectrometers (QE, QEplus, QE-HF, and QE-HF-X) [2]. We used TCGA RNA expression levels of the same genes obtained with Illumina HiSeq 4000 platform for the same biosamples to compare proteomic and transcriptomic data at the individual gene- and pathway activation levels. At the gene level, absolute gene expression values were compared, whereas the pathway-grade comparisons were made between the pathway activation levels (PALs) calculated using average sample-normalized transcriptomic and proteomic profiles. We observed remarkably different average correlations between the primary RNA- and protein expression data for different cancer types: Spearman Rho between 0.017 (*p* = 1.7 × 10^−13^) and 0.27 (*p* < 2.2 × 10^−16^). However, at the pathway level, we detected overall statistically significantly higher correlations: averaged Rho between 0.022 (*p* < 2.2 × 10^−16^) and 0.56 (*p* < 2.2 × 10^−16^). Thus, we conclude that data analysis at the PAL level enables obtaining results of a greater similarity when comparing high-throughput RNA and protein expression profiles.

## 2. Results

Intracellular molecular pathways include gene products participating in common molecular processes, i.e., in all major cellular events in health and disease. Traditionally, they are primarily classified as the metabolic, DNA repair, signaling, and cytoskeleton organization pathways [1]. Quantitative assessments of pathway activation levels (PALs) have given rise to next generation biomarkers in human biology that are, in many contexts, more accurate and robust than individual gene expression levels [13]. Herein, positive, zero, and negative PAL values mean upregulation, no changes, and downregulation of a molecular pathway, respectively [14]. Furthermore, the absolute value of PAL corresponds to the extent of a pathway differential regulation [13]. Thus, a higher PAL value reflects greater pathway activation and vice versa. For several experimental techniques regarding RNA expression analysis, PALs also showed less significant platform bias and batch effects than individual gene expression profiles [15]. In this study, we compared, for the first time, RNA and protein expression levels for the same biosamples at both the pathway- and individual gene activation levels. The biospecimens were human cancer tissue samples. Primary RNA and protein expression data were extracted from the TCGA and CPTAC project databases, respectively. The platform for RNA expression profiling was Illumina HiSeq 4000, and proteomic profiles were obtained using tandem mass (MS/MS) spectrometry conducted on Thermo QE series of mass spectrometers (QE, QEplus, QE-HF, and QE-HF-X) [2]. In total, we analyzed 755 paired transcriptomic/proteomic biosamples for seven human cancer types: breast invasive carcinoma (99, 13.11%), glioblastoma (98, 12.98%), hepatocellular carcinoma (87, 11.52%), lung adenocarcinoma (111, 14.70%), ovarian serous cystadenocarcinoma (119, 15.76%), pancreatic ductal adenocarcinoma (140, 18.54%), and uterine corpus endometrial carcinoma (101, 13.38%).

**Figure 1:**
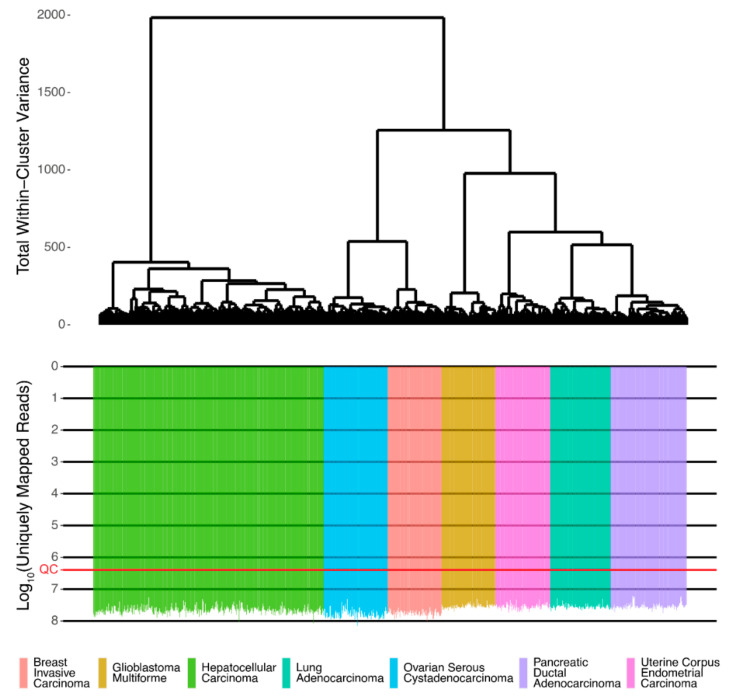
The dendrogram of hierarchical clustering for QC-passed RNA sequencing profiles of human tissues from TCGA. Gene expression data were used to calculate Euclidian distances between the samples. The color markers indicate the tissue types. The lower scale indicates the number of uniquely mapped reads. ‘QC’ marker denotes the quality control threshold of 2.5 million uniquely mapped reads.

Associations between protein and RNA expressions were assessed using Spearman Rho and Pearson R correlation coefficients for gene expression and pathway activation levels. The initial RNA sequencing profiles were screened and passed technical quality control metrics [16] (Figure 1).

**Figure 2:**
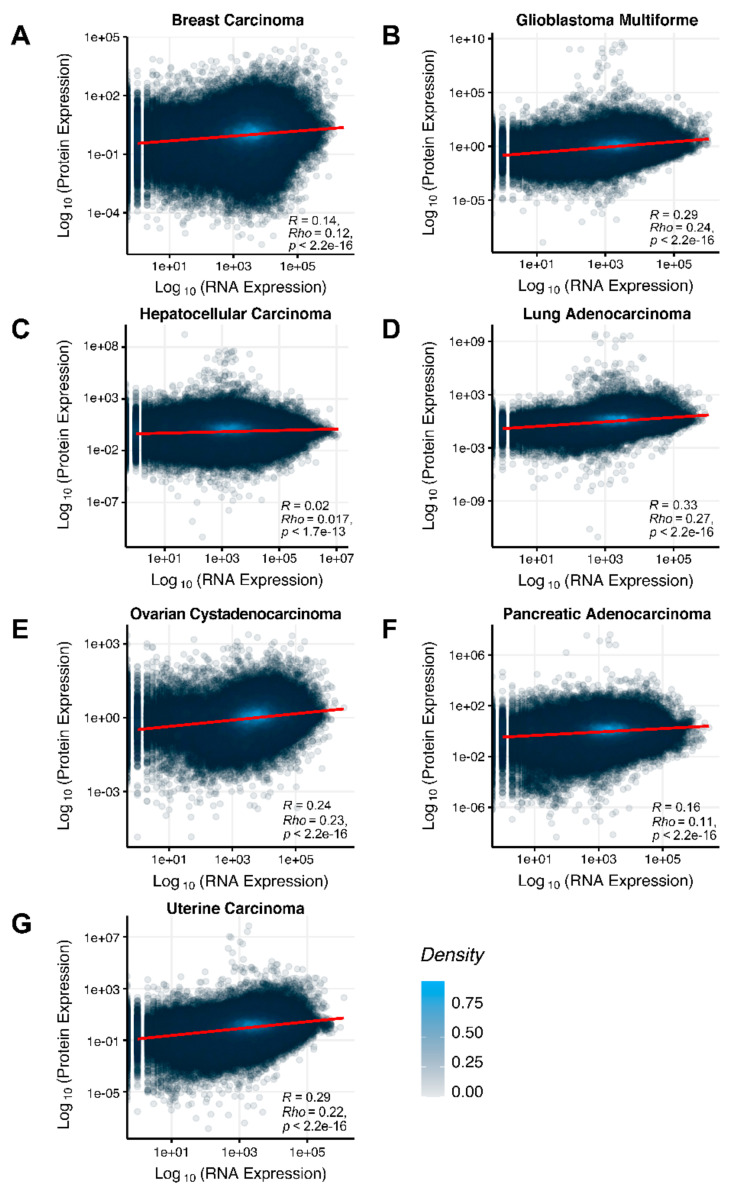
Gene-to-gene Spearman correlation between RNA and protein expression levels for an average biosample estimated across (**A**) Breast Invasive Carcinoma, (**B**) Glioblastoma Multiforme, (**C**) Hepatocellular Carcinoma, (**D**) Lung Adenocarcinoma, (**E**) Ovarian Serous Cystadenocarcinoma, (**F**) Pancreatic Ductal Adenocarcinoma and (**G**) Uterine Corpus Endometrial Carcinoma. Each dot represents a unique gene-sample pair.

We then assessed at the individual gene level Spearman correlations between RNA and protein expression levels in seven human cancer types. We obtained 0.17 mean Spearman correlation that varied from 0.017 for Hepatocellular Carcinoma till 0.27 for Lung Adenocarcinoma samples (Figure 2), which is in line with the previous results, i.e., ~0.16–0.35 correlation between the RNA- and proteome-based gene expression levels [6,7].

**Figure 3:**
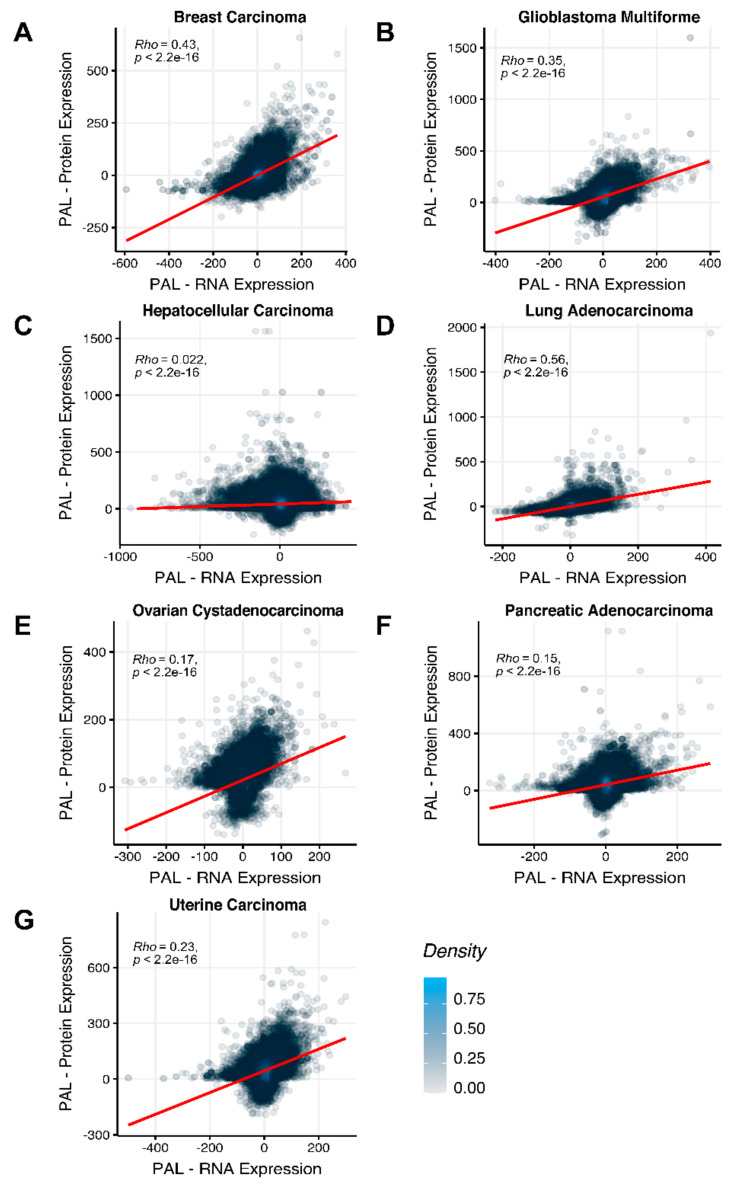
PAL-to-PAL Spearman correlation between RNA and protein expression levels for an average biosample estimated across (**A**) Breast Invasive Carcinoma, (**B**) Glioblastoma Multiforme, (**C**) Hepatocellular Carcinoma, (**D**) Lung Adenocarcinoma, (**E**) Ovarian Serous Cystadenocarcinoma, (**F**) Pancreatic Ductal Adenocarcinoma and (**G**) Uterine Corpus Endometrial Carcinoma. Each dot represents a unique gene-sample pair.

To compare RNA and protein expression data at the pathway level, we used the PAL approach for the same biosamples. We calculated Spearman correlations between PAL values for all tumor types under investigation (Figure 3). We observed 0.27 mean Spearman correlation between PAL values, which varied from 0.022 for Hepatocellular Carcinoma up to 0.56 for Lung Adenocarcinoma samples. Thus, we obtained 1.58-fold-change between the averaged gene-to-gene and PAL-to-PAL correlations.

**Figure 4:**
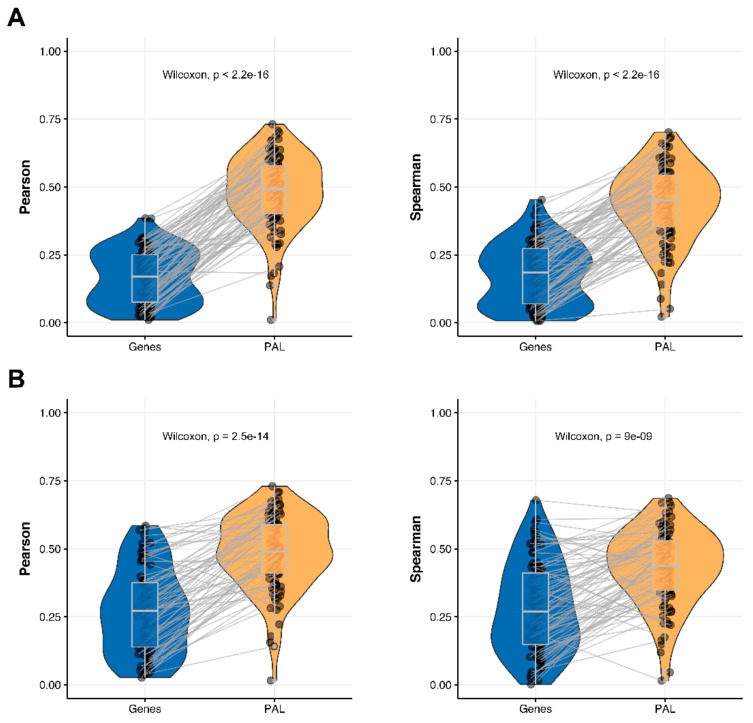
Paired gene-to-gene and PAL-to-PAL correlation between RNA and protein expression levels estimated within Breast Invasive Carcinoma biosamples using Pearson (*left*) and Spearman (*right*) correlation coefficients for (**A**) the total set of genes and molecular pathways; (**B**) the set of drug target genes and molecular pathways [17].

**Figure 5:**
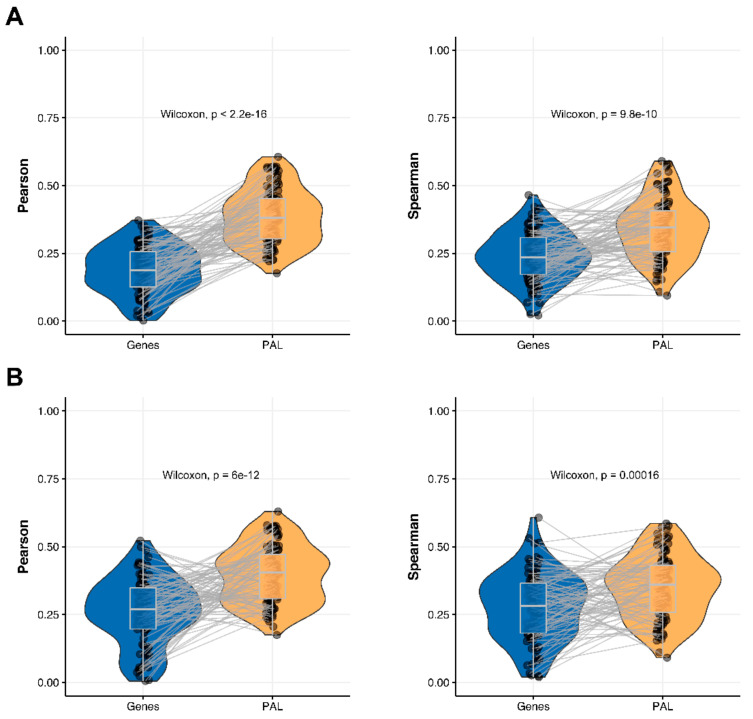
Paired gene-to-gene and PAL-to-PAL correlation between RNA and protein expression levels estimated within Glioblastoma Multiforme biosamples using Pearson (*left*) and Spearman (*right*) correlation coefficients for (**A**) the total set of genes and molecular pathways; (**B**) the set of drug target genes and molecular pathways [17].

**Figure 6:**
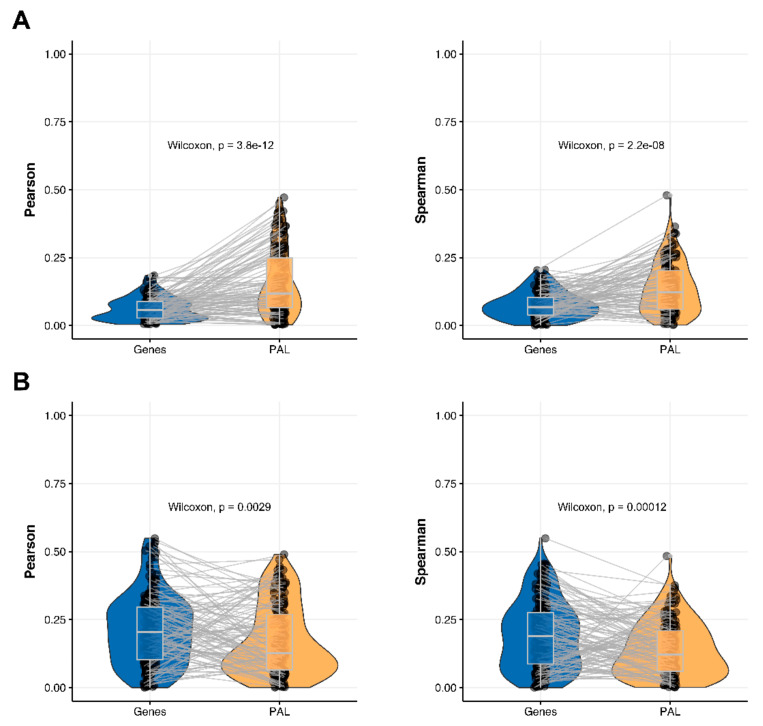
Paired gene-to-gene and PAL-to-PAL correlation between RNA and protein expression levels estimated within Hepatocellular Carcinoma biosamples using Pearson (*left*) and Spearman (*right*) correlation coefficients for (**A**) the total set of genes and molecular pathways; (**B**) the set of drug target genes and molecular pathways [17].

**Figure 7:**
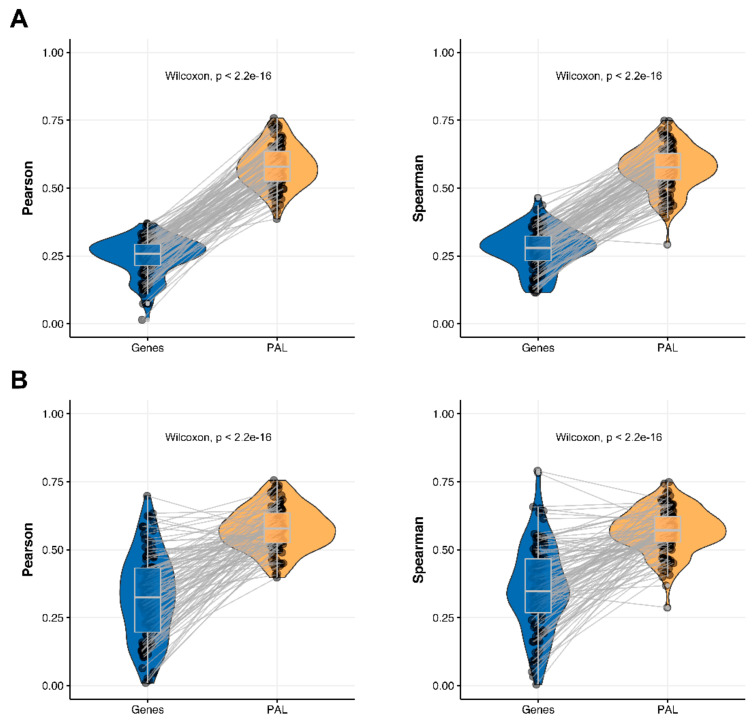
Paired gene-to-gene and PAL-to-PAL correlation between RNA and protein expression levels estimated within Lung Adenocarcinoma biosamples using Pearson (*left*) and Spearman (*right*) correlation coefficients for (**A**) the total set of genes and molecular pathways; (**B**) the set of drug target genes and molecular pathways [17].

**Figure 8:**
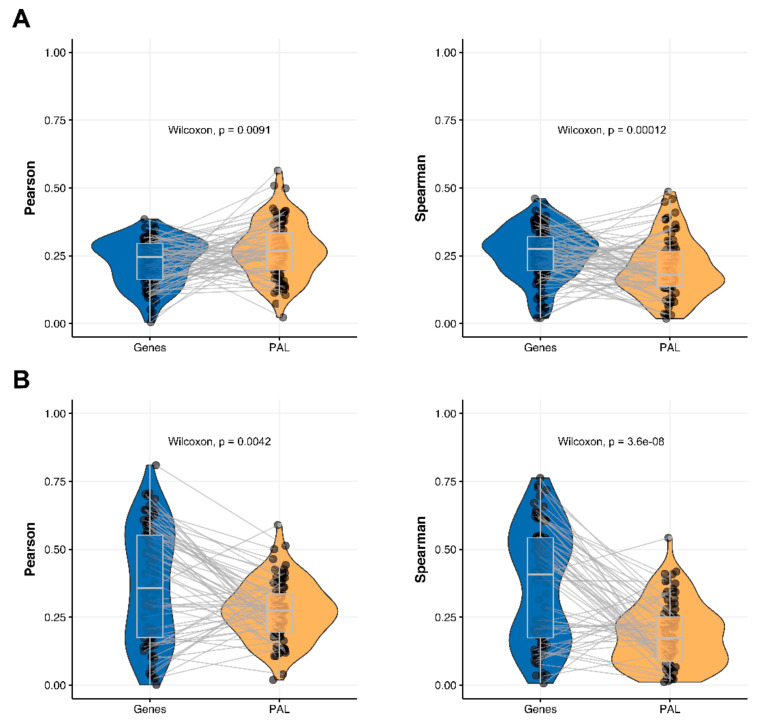
Paired gene-to-gene and PAL-to-PAL correlation between RNA and protein expression levels estimated within Ovarian Serous Cystadenocarcinoma biosamples using Pearson (*left*) and Spearman (*right*) correlation coefficients for (**A**) the total set of genes and molecular pathways; (**B**) the set of drug target genes and molecular pathways [17].

**Figure 9:**
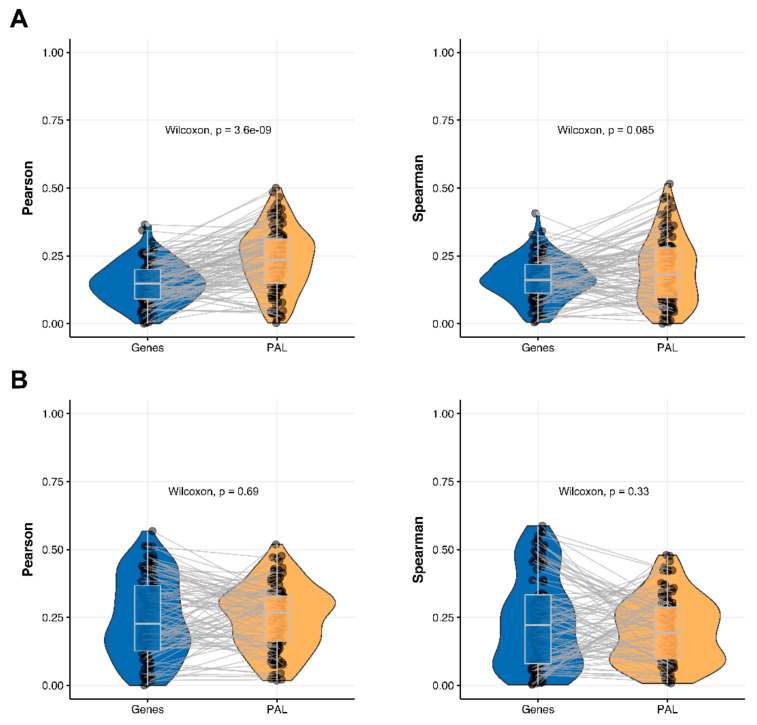
Paired gene-to-gene and PAL-to-PAL correlation between RNA and protein expression levels estimated within Pancreatic Ductal Adenocarcinoma biosamples using Pearson (*left*) and Spearman (*right*) correlation coefficients for (**A**) the total set of genes and molecular pathways; (**B**) the set of drug target genes and molecular pathways [17].

**Figure 10:**
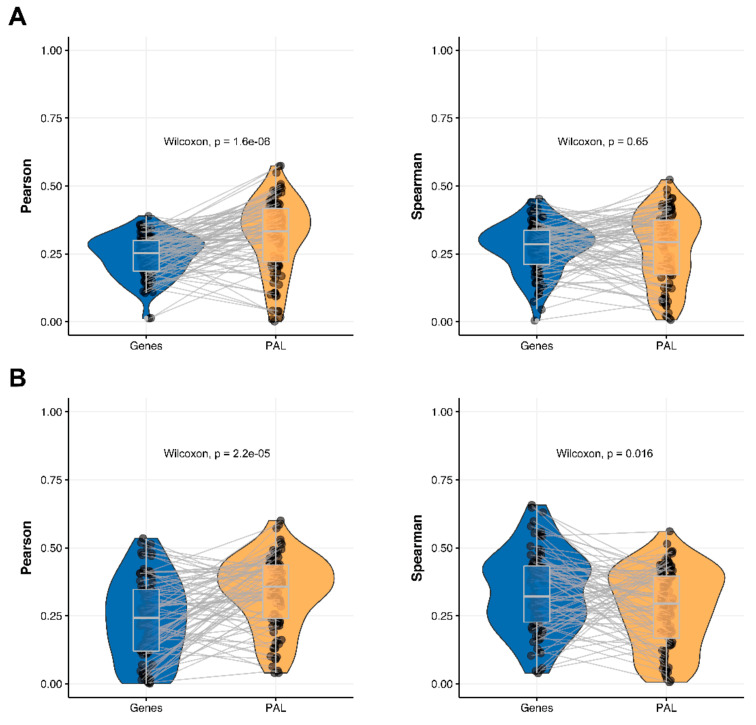
Paired gene-to-gene and PAL-to-PAL correlation between RNA and protein expression levels estimated within Uterine Corpus Endometrial Carcinoma biosamples using Pearson (*left*) and Spearman (*right*) correlation coefficients for (**A**) the total set of genes and molecular pathways; (**B**) the set of drug target genes and molecular pathways [17].

We then measured statistical significance of the differences between gene (transcriptome)-to-gene(proteome) vs. PAL (transcriptome)-to-PAL (proteome) correlations. Using Wilcoxon statistical test, we observed (Figure 4, Figure 5, Figure 6, Figure 7, Figure 8, Figure 9 and Figure 10) that both Pearson and Spearman correlations calculated for pathway activation levels were statistically significantly higher for the PAL level compared to the gene level in five out of seven cancer types investigated. In the remaining two cancer types, the results for Pearson and Spearman correlations were inconsistent or statistically not significant, as for ovarian serous cystadenocarcinoma (Figure 8) and uterine corpus endometrial carcinoma (Figure 10).

**Figure 11:**
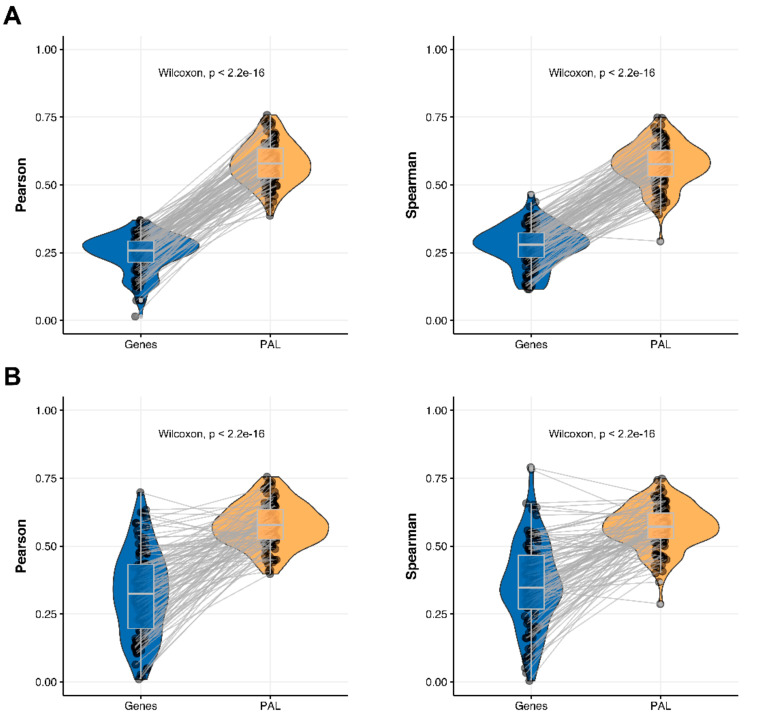
Paired gene-to-gene and PAL-to-PAL correlation between RNA and protein expression levels estimated within Lung Adenocarcinoma biosamples using Pearson (*left*) and Spearman (*right*) correlation coefficients for (**A**) the total set of genes and molecular pathways; (**B**) the set of drug target genes and molecular pathways [17].

We then performed this analysis for only 78 genes encoding molecular targets for the NCCN-recommended drugs in the seven cancer types considered in this study [17]. We compared 78-gene expression profiles and the profiles of molecular pathways including these genes at the RNA and protein levels. We observed that Pearson and Spearman correlations for pathway activation levels were statistically significantly higher compared to the single gene expression levels in the same four out of seven cancer types (Figure 4, Figure 5, Figure 9 and Figure 11). For the remaining three cancer types, i.e., hepatocellular carcinoma, ovarian serous cystadenocarcinoma, and uterine corpus endometrial carcinoma, Pearson and Spearman correlations showed poor statistical significance (Figure 6, Figure 8 and Figure 10).

**Figure 12:**
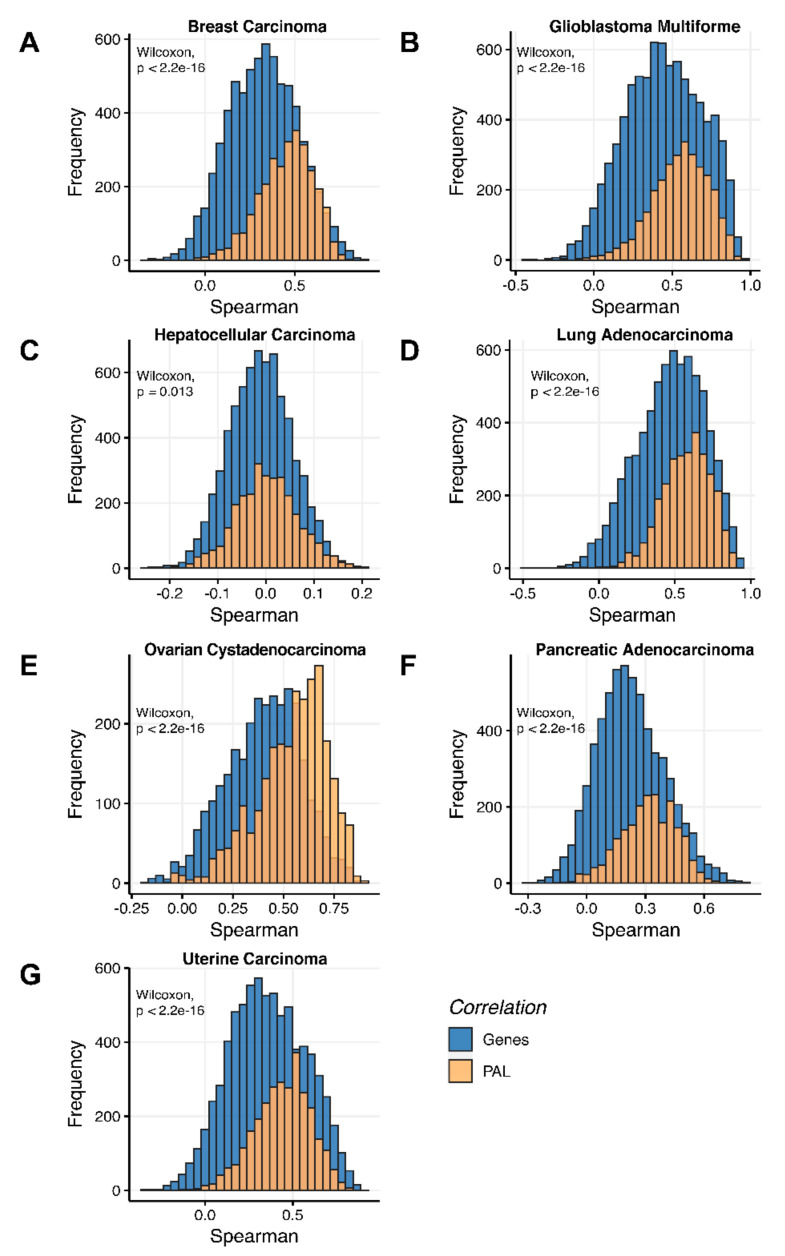
Gene-to-gene and PAL-to-PAL Spearman correlation between RNA and protein expression levels estimated by given gene or molecular pathway across (**A**) Breast Invasive Carcinoma, (**B**) Glioblastoma Multiforme, (**C**) Hepatocellular Carcinoma, (**D**) Lung Adenocarcinoma, (**E**) Ovarian Serous Cystadenocarcinoma, (**F**) Pancreatic Ductal Adenocarcinoma and (**G**) Uterine Corpus Endometrial Carcinoma.

**Figure 13:**
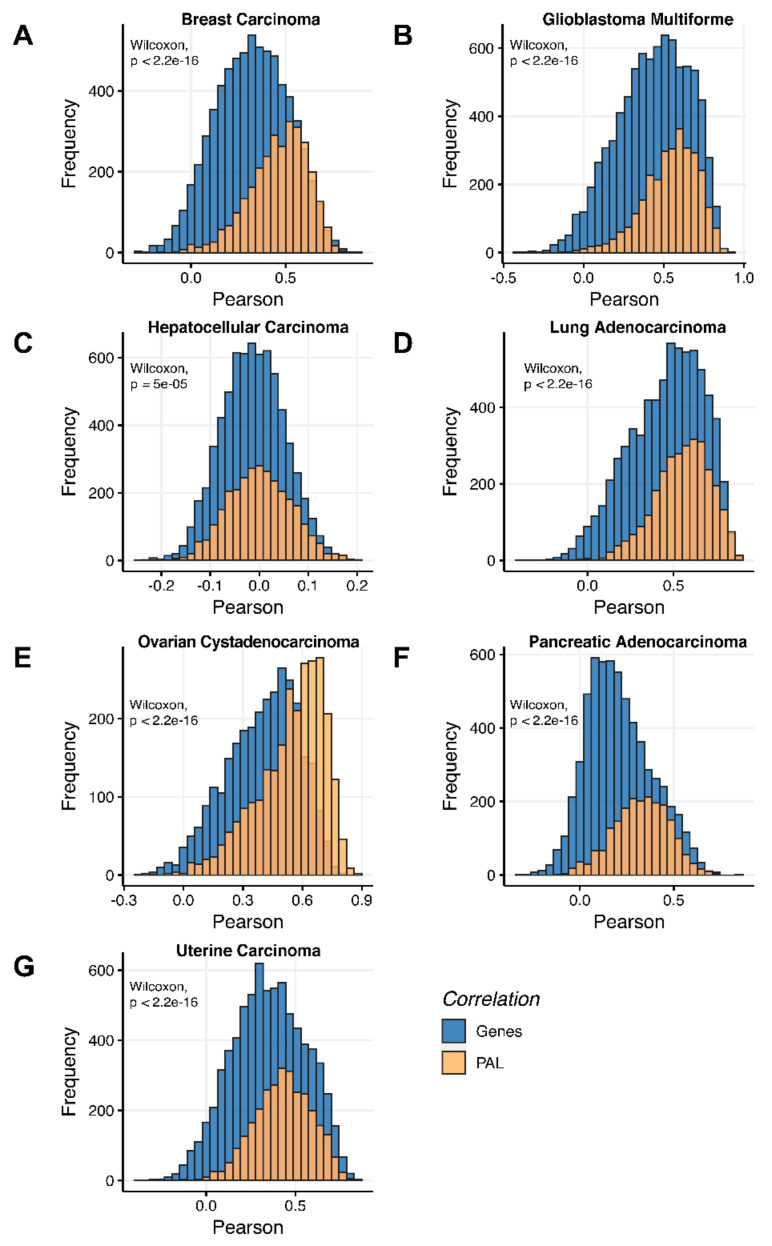
Gene-to-gene and PAL-to-PAL Pearson correlation between RNA and protein expression levels estimated given gene or molecular pathway across (**A**) Breast Invasive Carcinoma, (**B**) Glioblastoma Multiforme, (**C**) Hepatocellular Carcinoma, (**D**) Lung Adenocarcinoma, (**E**) Ovarian Serous Cystadenocarcinoma, (**F**) Pancreatic Ductal Adenocarcinoma and (**G**) Uterine Corpus Endometrial Carcinoma.

Additionally, we investigated how activation patterns of individual molecular pathways correlated between the different biosamples and compared this with the gene-specific patterns. We calculated Pearson and Spearman correlations for every gene and every molecular pathway among all biosamples of a given tumor type (Figure 12 and Figure 13). We observed that these correlations were statistically significantly higher for pathway activation levels, with the lowest statistical significance (highest *p* = 0.013) being observed for hepatocellular carcinoma.

Separately for each cancer type, we then analyzed the “top-10” of the most and the least correlated molecular pathways between RNA and protein expression data (see Table 1, Table 2, Table 3, Table 4, Table 5, Table 6 and Table 7 and Supplementary Table S1). Notably, such highly correlated pathways with regard to tumor infiltration, immune response, and regulation of DNA polymerase alpha, delta and epsilon activity were consistent among most tumor types (Spearman correlation 0.61–0.91).

**Figure 14:**
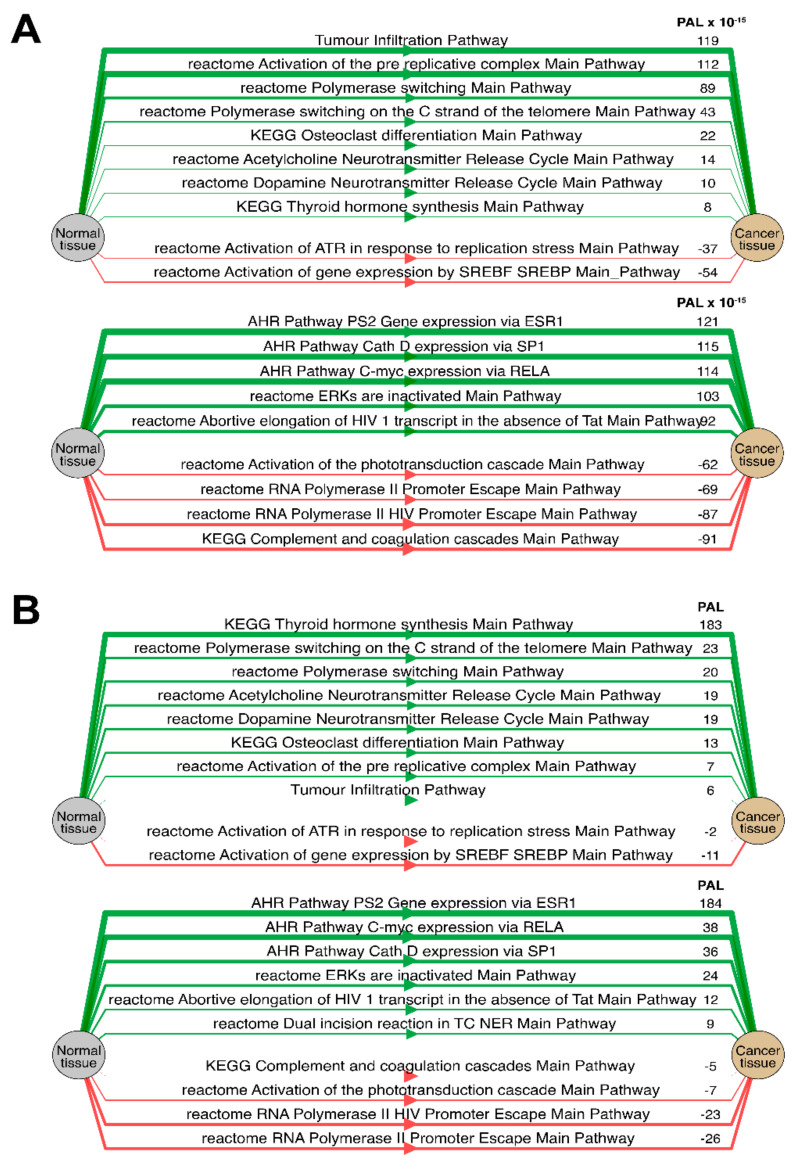
Diagram of activation (green) and inhibition (red) of top 10 of the most (*top*) and the least (*bottom*) correlated molecular pathways between RNA and protein expression for Lung Adenocarcinoma biosamples. Pathway activation level (PAL) independently estimated based on (**A**) protein levels and (**B**) RNA expression.

**Figure 15:**
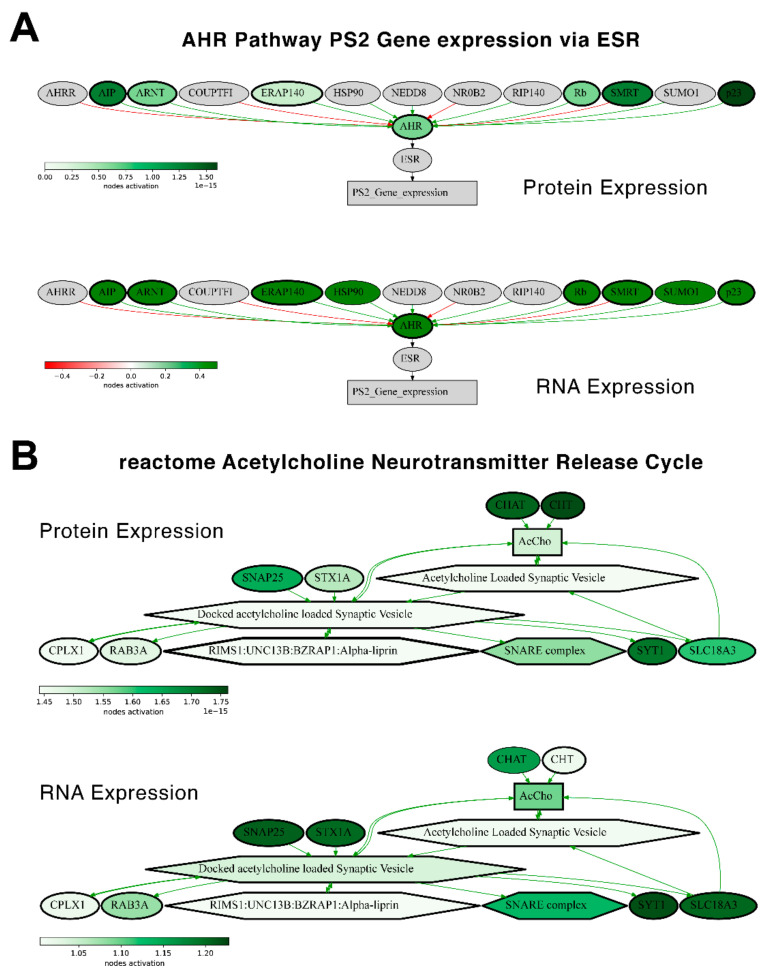
Molecular pathway activation maps estimated for protein expression (*top*) and RNA expression (*bottom*) of an averaged Lung Adenocarcinoma sample. Activation maps of (**A**) “AHR Pathway PS2 Gene expression via ESR” and (**B**) “reactome Acetylcholine Neurotransmitter Release Cycle Main Pathway” pathways. Genes and nodes that preserved a concordance of activation/inhibition between RNA and protein expression are bold-circled.

We then checked the consistencies of pathway activation schemes built using transcriptomic or proteomic data for the best and the least correlated molecular pathways. We calculated PAL levels for the “top-10” such pathways for Lung Adenocarcinoma biosamples (Figure 14) and compared activation charts for the chosen “AHR Pathway PS2 Gene expression via ESR” and “reactome Acetylcholine Neurotransmitter Release Cycle Main Pathway” pathways, which have the highest and the lowest correlations, respectively, for Lung Adenocarcinoma (Figure 15). Notably, all the pathways from both the best and the least correlated “top-10” group showed congruent activation patterns for RNAseq and proteomic data. Similarly, most of the components of the two molecular pathways that were considered more in-depth also showed congruent activation trends (Figure 15).

The number of gene products in a pathway theoretically may have an impact on the extent of the data aggregation effect [15] and, consequently, influence the gain of correlation in the gene-pathway comparisons. Therefore, we separately estimated gene-pathway correlations for the groups of bigger and smaller pathways including, respectively, at least 10, 20, and 40 gene products (Supplementary Figures S1–S7). We noticed, however, that the pathway size did not have any detectable impact on the pathway-level correlation gain.

Specifically, we obtained the following results for the individual cancer types under investigation.

### 2.1. Breast Invasive Carcinoma

**Figure 16:**
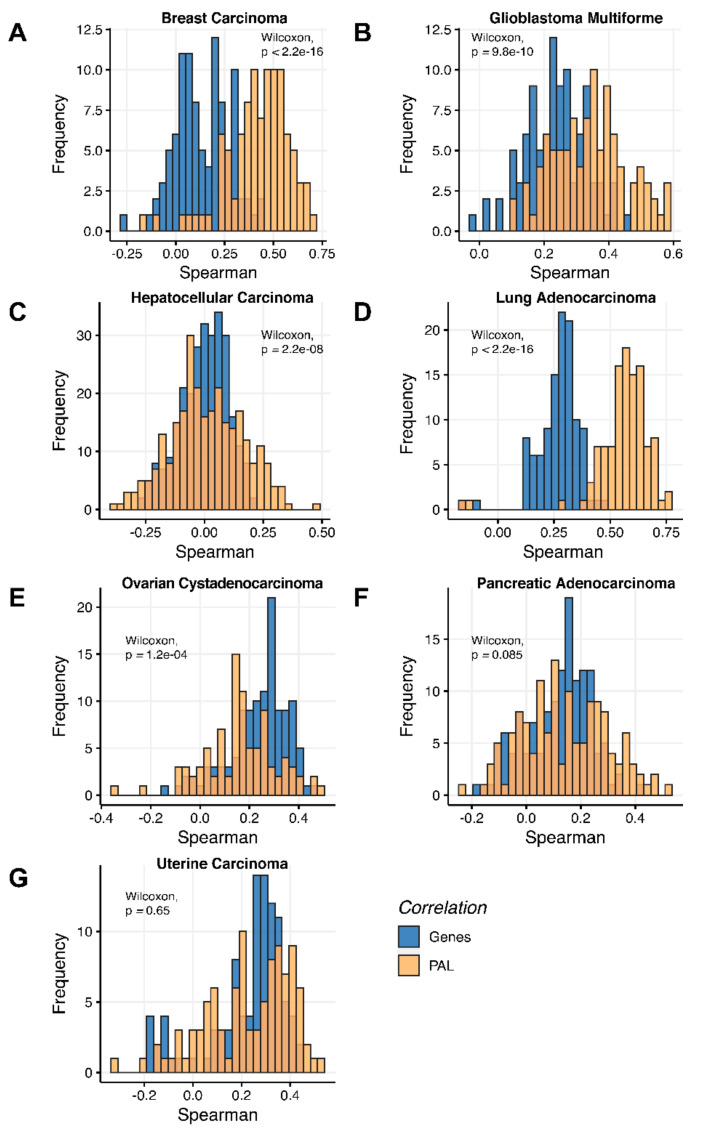
Gene-to-gene and PAL-to-PAL Spearman correlation between RNA and protein expression levels estimated by individual samples across (**A**) Breast Invasive Carcinoma, (**B**) Glioblastoma Multiforme, (**C**) Hepatocellular Carcinoma, (**D**) Lung Adenocarcinoma, (**E**) Ovarian Serous Cystadenocarcinoma, (**F**) Pancreatic Ductal Adenocarcinoma and (**G**) Uterine Corpus Endometrial Carcinoma.

**Figure 17:**
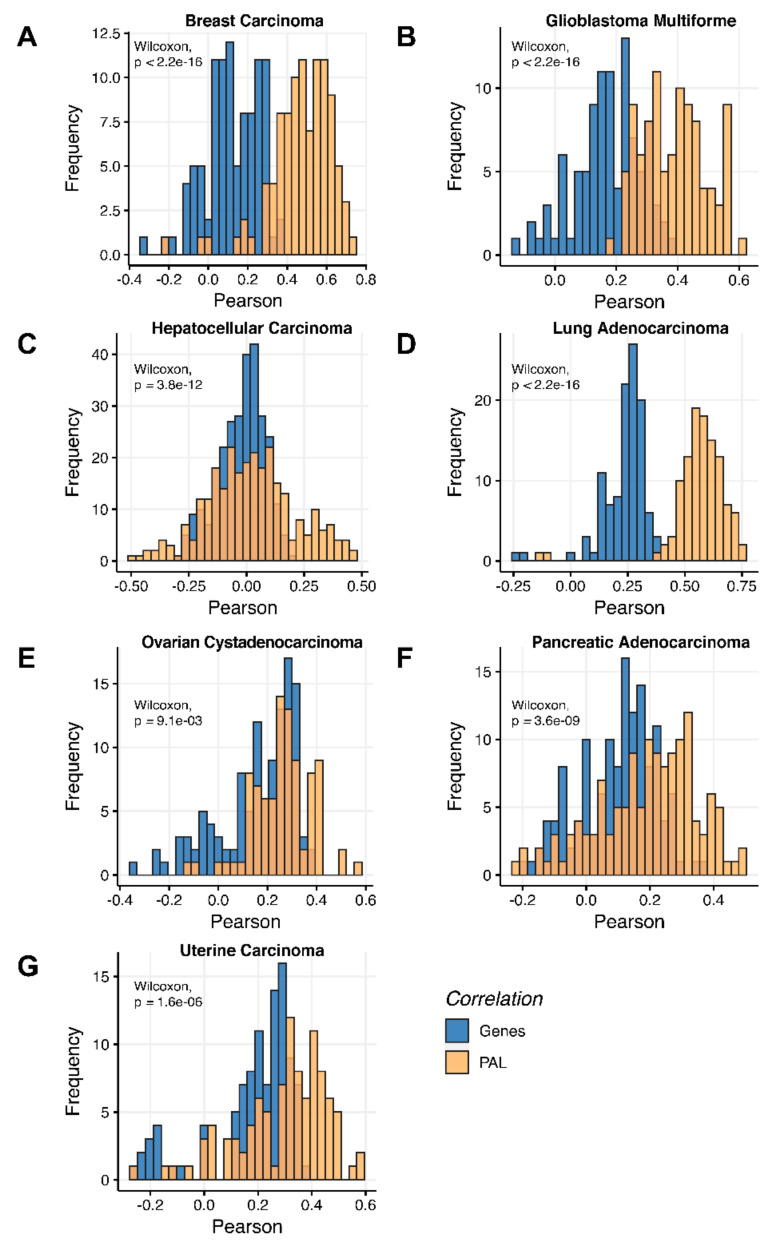
Gene-to-gene and PAL-to-PAL Pearson correlation between RNA and protein expression levels estimated by individual samples across (**A**) Breast Invasive Carcinoma, (**B**) Glioblastoma Multiforme, (**C**) Hepatocellular Carcinoma, (**D**) Lung Adenocarcinoma, (**E**) Ovarian Serous Cystadenocarcinoma, (**F**) Pancreatic Ductal Adenocarcinoma and (**G**) Uterine Corpus Endometrial Carcinoma.

The Pearson and Spearman gene-to-gene correlations between RNA and protein expression for an averaged biosample (Figure 2A) were 0.14 and 0.12, respectively, while on the PAL-to-PAL level (Figure 3A), they were 0.47 and 0.43, respectively. Thus, data analysis at the pathway activation levels resulted in ~3.5 times higher transcriptome-proteome correlation compared to the gene level, which was statistically significant (Wilcoxon *p* < 2.2 × 10^−16^); see Figure 4A, Figure 16A and Figure 17A. This difference also remained significant (*p* < 2.2 × 10^−16^) for drug target genes and molecular pathways (Figure 4B).

Pearson and Spearman correlations for individual genes or molecular pathways were statistically significantly higher for pathway activation levels (Wilcoxon, both *p* < 2.2 × 10^−16^, Figure 12A and Figure 13A).

The “top-10” most strongly and weakly correlated molecular pathways are shown on Table 1.

### 2.2. Glioblastoma Multiforme

The Pearson and Spearman gene-to-gene correlations for an average biosample (Figure 2B) were 0.29 and 0.24, respectively, and PAL-to-PAL correlations (Figure 3B) were 0.39 and 0.35, respectively. Thus, in this case, we detected ~1.5 times pathway-level gain of correlation, which was statistically significant (*p* < 2.2 × 10^−16^ and *p* < 10^−9^ for Pearson and Spearman correlations, respectively); see Figure 5A, Figure 16B and Figure 17B. This difference also remained statistically significant for the cancer drug-targeted genes and molecular pathways (Figure 5B).

Pearson and Spearman correlations for individual genes or pathways were statistically significantly higher for pathway activation levels (Wilcoxon, both *p* < 2.2 × 10^−16^, Figure 12B and Figure 13B).

The “top-10” of the most and the least correlated pathways are shown on Table 2.

### 2.3. Hepatocellular Carcinoma

The Pearson and Spearman gene-to-gene correlations for an average biosample (Figure 2C) were 0.02 and 0.017, respectively, and PAL-to-PAL correlations (Figure 3C) were 0.061 and 0.022, respectively. This corresponded to ~3 times pathway-level gain for the Pearson correlation (*p* < 2.2 × 10^−16^), whereas the fold-change for the Spearman correlation was only ~1.2, which was also statistically significant (*p* = 2.2 × 10^−8^); see Figure 6A, Figure 16C and Figure 17C. This difference, however, had the opposite effect on cancer drug targeted genes and molecular pathways (Figure 6B).

The Pearson and Spearman correlations for individual genes or molecular pathways were statistically significantly higher for pathway activation levels (Wilcoxon, *p* = 5 × 10^−5^ and *p* = 0.013, respectively); see Figure 12C and Figure 13C.

The “top-10” of the most and the least correlated molecular pathways are shown on Table 3.

### 2.4. Lung Adenocarcinoma

The Pearson and Spearman gene-to-gene correlations for an average biosample (Figure 2D) were 0.33 and 0.27, respectively, and PAL-to-PAL correlations (Figure 3D) were 0.58 and 0.56, respectively. Thus, the detected pathway-level gain of correlation was ~2 times (*p* < 2.2 × 10^−16^); see Figure 7A, Figure 16D and Figure 17D. The correlation gain also remained statistically significant for cancer drug-targeted genes and molecular pathways (Figure 7B).

Pearson and Spearman correlations for individual genes or molecular pathways were statistically significantly higher for pathway activation levels (Wilcoxon, both *p* < 2.2 × 10^−16^, Figure 12D and Figure 13D).

The “top-10” of the most and the least correlated molecular pathways are shown on Table 4.

We also compared PAL levels for “top-10” lists of the most and the least correlated molecular pathways in Lung Adenocarcinoma biosamples (Figure 14) for the transcriptomic and proteomic data. All of the most and the least correlated pathways showed common activation or inhibition trends for the RNA and protein expression data.

Similarly, the activation charts for “AHR Pathway PS2 Gene expression via ESR” and “reactome Acetylcholine Neurotransmitter Release Cycle Main Pathway” pathways were compared (Figure 15). Similarly, most of the components in the two considered molecular pathways were also congruently activated.

### 2.5. Ovarian Serous Cystadenocarcinoma

The Pearson and Spearman gene-to-gene correlations for an average biosample (Figure 2E) were 0.24 and 0.23, respectively, and PAL-to-PAL correlations (Figure 3E) were 0.25 and 0.17, respectively. In this case, no pathway-level correlation gain was detected (Pearson ~1; Spearman ~0.74 times) either for drug target genes or molecular pathways (Figure 8B).

The Pearson and Spearman correlations for individual genes or molecular pathways were statistically significantly higher for pathway activation levels (Wilcoxon, both *p* < 2.2 × 10^−16^); see Figure 12E and Figure 13E.

The “top-10” of the most and the least correlated molecular pathways between RNA and protein expression levels are shown on Table 5.

### 2.6. Pancreatic Ductal Adenocarcinoma

Pearson and Spearman gene-to-gene correlations for an average biosample (Figure 2F) were 0.16 and 0.11, respectively, and PAL-to-PAL correlations (Figure 3F) were 0.19 and 0.15, respectively. This suggests ~1.4 times pathway-level gain of correlation which was statistically significant only for Spearman (*p* = 3.6 × 10^−9^) correlation; see Figure 5F, Figure 15A and Figure 16F. Differences between Spearman correlations were also statistically significant for drug target genes and molecular pathways (Figure 9B).

The Pearson and Spearman correlations for individual genes or molecular pathways were statistically significantly higher for pathway activation levels (Wilcoxon, both *p* < 2.2 × 10^−16^); see Figure 12F and Figure 13F.

The “top-10” of the most and the least correlated molecular pathways are shown in Table 6.

### 2.7. Uterine Corpus Endometrial Carcinoma

The Pearson and Spearman gene-to-gene correlations for an average biosample (Figure 2G) were 0.29 and 0.22, respectively, and PAL-to-PAL correlations (Figure 3G) were 0.29 and 0.23, respectively. In this case, correlations remained essentially the same for both gene- and pathway levels; see Figure 10A, Figure 16G and Figure 17G. The same trend was also seen for drug target genes and pathways (Figure 10B).

Pearson and Spearman correlations for individual genes or molecular pathways were statistically significantly higher for pathway activation levels (Wilcoxon, both *p* < 2.2 × 10^−16^, Figure 12G and Figure 13G).

The “top-10” of the most and the least correlated molecular pathways between RNA and protein expression levels are shown on Table 7.

## 3. Discussion

Quantitative gene expression profiles at the mRNA and protein levels are fundamentally different because of differential mRNA and protein stability patterns, epigenetic and protein translation regulatory mechanisms [18] and technical differences in the screening methods [19]. However, there is an overall correlation between the quantitative gene expression profiles that was detected for various organisms and cell types [18].

In this study we aimed to compare correlations of RNA- and protein-level gene expression data at the single gene and molecular pathway levels.

At the gene level, the correlations observed were congruent with the literature data (e.g., we observed ~0.23–0.24 correlations for ovarian cancer at the gene level, compared to ~0.3 in the previous report [7], or ~0.24–0.29 for glioblastoma in this study compared to previously observed ~0.15 [6]).

On the other hand, we detected remarkably different correlations between RNA expression and proteomic data on both gene- and pathway levels between the different tumor types investigated simultaneously in TCGA and CPTAC projects. For example, the lowest correlation could be observed for hepatocellular carcinoma (0.017–0.06), whereas the biggest was detected for lung adenocarcinoma (0.33–0.58).

Such dramatic difference from the first view could be explained by vulnerabilities of the experimental expression platforms to the nature of the tested biospecimens. However, some biological mechanisms could also be involved, e.g., tumor type-specific pH alteration, which can lead to dramatic differences in the repertoire of translated proteins while not strongly affecting RNA transcription [20]. 

In any case, we noticed that in five out of seven cancer types tested (glioblastoma, breast, liver, lung, pancreatic cancers), the expression data analysis at the pathway level was beneficial in terms of improving the correlation between the quantitative mRNA and protein data for the same biospecimens. In the two remaining tumor types (endometrial and ovarian cancers), we observed no pathway-level gain of correlation between mRNA and proteomic data. These results did not depend on the pathway size, as they were reproduced here with pathways of any size, and with pathways with 10-, 20-, and 40-participant size limits.

Similarly, we observed a gain of correlation between mRNA and protein expression in four out of seven cancer types in a correlation analysis of cancer drug-targeted genes and molecular pathways.

## 4. Materials and Methods

### 4.1. Processing of RNA Sequencing Data

Ensembl gene IDs were converted to HGNC gene symbols according to the Complete HGNC dataset (https://www.genenames.org, accessed on 14 November 2021. Overall, expression levels were established for 36,596 annotated genes with HGNC identifiers. Additionally, ‘1’ was added to all raw gene counts prior to cluster analyses to avoid zero expression values, following the recommendation by Dillies et al. [21]. The gene expression data were merged into a single dataset and preprocessed using *DESeq2* [22] as a normalization method. Hierarchical clustering was performed using R *“ward.D2”* method. We used a threshold of 2.5 M uniquely mapped reads for QC of RNA sequencing data (Figure 1), as this was found to be effective for marking samples with low-quality values of other QC metrics, e.g., the proportion of genomic counts, high rate of mismatches, number of reads spanning splice junction, a high percentage of ribosomal counts [16].

### 4.2. Publicly Available Transcriptomic and Proteomic Paired Data

We used paired transcriptomic and proteomic profiles obtained from The Cancer Genome Atlas (TCGA) Data Portal [23] and Clinical Proteomic Tumor Analysis Consortium (CPTAC) Repository [24]. In total, 755 paired transcriptomic and proteomic biosamples were analyzed for seven cancer types: Breast Invasive Carcinoma (99, 13.11%), Glioblastoma Multiforme (98, 12.98%), Hepatocellular Carcinoma (87, 11.52%), Lung Adenocarcinoma (111, 14.70%), Ovarian Serous Cystadenocarcinoma (119, 15.76%), Pancreatic Ductal Adenocarcinoma (140, 18.54%), and Uterine Corpus Endometrial Carcinoma (101, 13.38%).

### 4.3. Molecular Pathway Annotation and Activation Scoring

In this study we used a publicly available collection of molecular pathways extracted from Biocarta version 1.2 [25], Qiagen Pathway Central [26], Kyoto Encyclopedia of Genes and Genomes (KEGG) [1], NCI database version 1.2 [27], and Reactome version 1.3 [13] databases, and algorithmically annotated for molecular functions of pathway components and nodes [28]. Using the Oncobox bioinformatics platform [15,16] we calculated pathway activation levels (PALs) for a total of 1611 molecular pathways containing 10 or more gene products. For PAL calculations, each sample expression profile was normalized on mean geometrical levels of RNA or protein expression for all samples in the dataset under analysis.

The PAL approach considers the impact of each gene product on overall molecular pathway activation [29,30], the PAL value for a pathway p a given sample is calculated as follows:

$$PAL_{p}=\sum_{n}ARR_{np}\cdot BTIF_{n}\cdot ln\left(CNR_{n}\right)$$

where $CNR_{n}$ (*case-to-normal ratio)* is the ratio of gene n expression level in the sample under investigation to the mean geometrical gene n expression level in the group of control samples. The Boolean flag $BTIF_{n}$ *(beyond tolerance interval flag*) is zero when the $CNR_{n}$ value has not passed the significance criterion: when the difference with the control group of samples is not significant, where p>0.05 $ARR_{n,p}$ *(activator/repressor role of gene n in pathway p)* is the discrete value that equals to −1 when gene product n is a repressor of pathway *p*; 1, when gene product n is an activator of pathway *p*; 0, when gene product n has both activities of an activator and of a repressor of pathway *p*; 0.5 and −0.5, respectively, when gene product n is rather an activator or repressor of pathway p.

## 5. Conclusions

In this study, we aimed to compare correlations among RNA- and protein-level gene expression data at the single gene and molecular pathway levels. We detected remarkably different correlations between RNA expression and proteomic data on both gene- and pathway levels among the different tumor types investigated simultaneously in TCGA and CPTAC projects. For example, the lowest correlation was observed for hepatocellular carcinoma (0.017–0.06), whereas the biggest was detected for lung adenocarcinoma (0.33–0.58). This dramatic difference could be due to the vulnerabilities of the experimental expression platforms to the nature of the tested biospecimens. However, some biological mechanisms may also be involved, e.g., tumor type-specific pH alteration, which can lead to dramatic differences in the repertoire of translated proteins while not strongly affecting RNA transcription [20]. We also demonstrated that the assessment of pathway activation levels based on transcriptomic data produces largely congruent profiles with those for the proteomic profiles. Our results evidence that the pathway level of transcriptomic data analysis can be advantageous compared to the single-gene level because it can statistically significantly improve correlations among RNA- and proteomic data in most of the tested cases.

## Figures and Tables

**Table 1:** “Top-10” of the most and the least correlated molecular pathways between RNA and protein expression data calculated for Breast Invasive Carcinoma biosamples.

Pathway	Pearson	Spearman
Ephrin-mediated Signaling Events During Cell Adhesion	0.73	0.77
Guanosine nucleotides ide novoi biosynthesis	0.74	0.76
Reactome Polymerase switching Main Pathway	0.75	0.74
Reactome Polymerase switching on the C strand of the telomere Main Pathway	0.75	0.74
NCI Aurora B signaling Main Pathway	0.71	0.73
KEGG Bacterial invasion of epithelial cells Main Pathway	0.71	0.72
NAD ide novoi biosynthesis	0.74	0.72
Development of Immune_Synapse	0.70	0.71
KEGG Osteoclast differentiation Main Pathway	0.72	0.71
Tumor Infiltration Pathway	0.72	0.71
KEGG PPAR signaling Main Pathway	0.13	0.10
KEGG Complement and coagulation cascades Main Pathway	0.14	0.07
Reactome regulation of FZD by ubiquitination Main Pathway	0.02	0.07
Biocarta induction of apoptosis through dr3 and dr4 5 death receptors Main Pathway	0.13	0.05
reactome ABCA transporters in lipid homeostasis Main Pathway	0.14	0.05
biocarta induction of apoptosis through dr3 and dr4 5 death receptors Pathway (apoptosis)	0.12	0.05
reactome Intrinsic Pathway Main Pathway	−0.02	0.03
reactome Sphingolipid de novo biosynthesis Main Pathway	−0.04	0.00
NCI N cadherin signaling events Pathway (myoblast differentiation)	−0.02	0.00
biocarta multi step regulation of transcription by pitx2 Main Pathway	0.04	−0.03

**Table 2:** “Top-10” of the most and the least correlated molecular pathways between RNA and protein expression data calculated for Glioblastoma Multiforme biosamples.

Pathway	Pearson	Spearman
Tumor Infiltration Pathway	0.89	0.91
reactome temp Immunoregulatory interactions between a Lymphoid and a Nonlymphoid cell	0.85	0.89
KEGG Legionellosis Main Pathway	0.83	0.89
NCI BCR signaling Main Pathway	0.84	0.88
KEGG Osteoclast differentiation Main Pathway	0.83	0.88
FCGR3A-mediated phagocytosis	0.85	0.88
Development of Immune Synapse	0.84	0.87
reactome Dissolution of Fibrin Clot Main Pathway	0.81	0.86
reactome Activation of the pre replicative complex Main Pathway	0.85	0.85
KEGG Lysosome Main Pathway	0.84	0.85
reactome Synthesis secretion and inactivation of Glucose dependent Insulinotropic Polypeptide GIP Main Pathway	0.18	0.19
reactome Pre NOTCH Processing in Golgi Main Pathway	0.19	0.18
reactome EPH ephrin mediated repulsion of cells Main Pathway	0.17	0.17
reactome Degradation of beta catenin by the destruction complex Main Pathway	0.18	0.16
TCA cycle	0.21	0.16
NCI Signaling events mediated by PRL Main Pathway	0.06	0.11
reactome Activation of the phototransduction cascade Main Pathway	0.06	0.08
NCI N cadherin signaling events Pathway (myoblast differentiation)	0.05	0.06
KEGG GABAergic synapse Main Pathway	0.06	0.02
reactome regulation of FZD by ubiquitination Main Pathway	−0.04	−0.05

**Table 3:** “Top-10” of the most and the least correlated molecular pathways between RNA and protein expression data calculated for Hepatocellular Carcinoma biosamples.

Pathway	Pearson	Spearman
Tumor Infiltration Pathway	0.89	0.91
biocarta the co stimulatory signal during t cell activation Pathway (T cell activation)	0.18	0.20
reactome Integrin cell surface interactions Main Pathway	0.15	0.19
Ras Pathway Apoptosis	0.16	0.19
IL-2 Pathway	0.15	0.18
VEGF Pathway	0.16	0.17
NCI Beta3 integrin cell surface interactions Main Pathway	0.11	0.16
NCI Beta1 integrin cell surface interactions Main Pathway	0.13	0.16
reactome Downstream TCR signaling Main Pathway	0.13	0.16
biocarta lck and fyn tyrosine kinases in initiation of tcr activation Main Pathway	0.16	0.15
NCI EPHA forward signaling Pathway (cell adhesion)	0.18	0.15
reactome Inhibition of voltage gated Ca_2_ channels via Gbeta gamma subunits Main Pathway	−0.07	−0.12
reactome Hyaluronan uptake and degradation Main Pathway	−0.09	−0.13
NCI N cadherin signaling events Pathway (myoblast differentiation)	−0.12	−0.13
NCI AP 1 transcription factor network Main Pathway	−0.11	−0.13
Akt Pathway Regulation by GH	−0.13	−0.14
chondroitin sulfate biosynthesis	−0.15	−0.14
IGF1R Signaling Pathway Apoptosis	−0.13	−0.15
HGF Pathway Cell Cycle Progression	−0.12	−0.15
reactome Detoxification of Reactive Oxygen Species Main Pathway	−0.15	−0.15
biocarta mapkinase signaling Main Pathway	−0.14	−0.16

**Table 4:** “Top-10” of the most and the least correlated molecular pathways between RNA and protein expression data calculated for Lung Adenocarcinoma biosamples.

Pathway	Pearson	Spearman
reactome Acetylcholine Neurotransmitter Release Cycle Main Pathway	0.88	0.90
Tumor Infiltration Pathway	0.83	0.89
reactome Dopamine Neurotransmitter Release Cycle Main Pathway	0.83	0.89
KEGG Thyroid hormone synthesis Main Pathway	0.64	0.87
reactome Activation of the pre replicative complex Main Pathway	0.86	0.86
reactome Activation of ATR in response to replication stress Main Pathway	0.86	0.86
KEGG Osteoclast differentiation Main Pathway	0.87	0.85
reactome Polymerase switching Main Pathway	0.84	0.85
reactome Polymerase switching on the C strand of the telomere Main Pathway	0.84	0.85
reactome Activation of gene expression by SREBF SREBP Main Pathway	0.80	0.84
reactome RNA Polymerase II HIV Promoter Escape Main Pathway	0.18	0.21
reactome RNA Polymerase II Promoter Escape Main Pathway	0.18	0.21
KEGG Complement and coagulation cascades Main Pathway	0.20	0.18
reactome Activation of the phototransduction cascade Main Pathway	0.12	0.17
AHR Pathway Cath D expression via SP1	0.32	0.16
reactome Abortive elongation of HIV 1 transcript in the absence of Tat Main Pathway	0.17	0.16
reactome Dual incision reaction in TC NER Main Pathway	0.09	0.13
AHR Pathway C-myc expression via RELA	0.31	0.12
reactome ERKs are inactivated Main Pathway	0.15	0.10
AHR Pathway PS2 Gene expression via ESR1	0.26	0.09

**Table 5:** “Top-10” of the most and the least correlated molecular pathways for RNA and protein expression levels calculated for Ovarian Serous Cystadenocarcinoma biosamples.

Pathway	Pearson	Spearman
KEGG Aldosterone regulated sodium reabsorption Main Pathway	0.69	0.90
Tumor Infiltration Pathway	0.83	0.87
biocarta lck and fyn tyrosine kinases in initiation of tcr activation Main Pathway	0.73	0.84
biocarta the co stimulatory signal during t cell activation Pathway (T cell activation)	0.72	0.83
HGF Pathway Cell Cycle Progression	0.80	0.83
TRAF Pathway Direct Antimicrobial Response and Cell-Mediated Immunity and Apoptosis of Host Cell	0.77	0.82
reactome Interleukin 2 signaling Main Pathway	0.76	0.82
NAD ide novoi biosynthesis	0.72	0.81
KEGG Glycosaminoglycan biosynthesis keratan sulfate Main Pathway	0.73	0.81
biocarta keratinocyte differentiation Main Pathway	0.75	0.80
reactome ADP signaling through P2Y purinoceptor 12 Main Pathway	0.21	0.16
KEGG Alcoholism Main Pathway	0.13	0.08
reactome APC Cdc20 mediated degradation of Nek2A Main Pathway	0.06	0.05
reactome Activation of G protein gated Potassium channels Main Pathway	0.10	0.00
reactome Inhibition of voltage gated Ca_2_ channels via Gbeta gamma subunits Main Pathway	0.10	0.00
reactome Presynaptic function of Kainate receptors Main Pathway	0.10	0.00
reactome Prostacyclin signaling through prostacyclin receptor Main Pathway	0.12	−0.01
reactome ABCA transporters in lipid homeostasis Main Pathway	−0.03	−0.02
reactome IRAK2 mediated activation of TAK1 complex Main Pathway	0.02	−0.04
reactome IRAK2 mediated activation of TAK1 complex upon TLR7 8 or 9 stimulation Main Pathway	0.02	−0.04

**Table 6:** “Top-10” of the most and the least correlated molecular pathways between RNA and protein expression levels calculated for Pancreatic Ductal Adenocarcinoma biosamples.

Pathway	Pearson	Spearman
KEGG Histidine metabolism Main Pathway	0.63	0.62
L-kynurenine degradation	0.61	0.61
NCI Beta5 beta6 beta7 and beta8 integrin cell surface interactions Main Pathway	0.59	0.58
Extracellular Matrix Remodeling during Adhesion	0.60	0.57
NCI Signaling events mediated by Hepatocyte Growth Factor Receptor c Met Pathway (positive regulation of tyrosine phosphorylation of STAT protein)	0.58	0.57
biocarta t cell receptor signaling Main Pathway	0.57	0.56
KEGG Synthesis and degradation of ketone bodies Main Pathway	0.56	0.56
biocarta repression of pain sensation by the transcriptional regulator dream Main Pathway	0.51	0.55
KEGG Glycosphingolipid biosynthesis ganglio series Main Pathway	0.52	0.54
Tumor Infiltration Pathway	0.63	0.54
Akt Signaling Pathway NFAT degradation	0.04	0.05
biocarta how does salmonella hijack a cell Pathway (lamellipodium assembly)	0.07	0.05
biocarta role of pi3k subunit p85 in regulation of actin organization and cell migration Main Pathway	0.08	0.05
KEGG Bladder cancer Main Pathway	0.11	0.05
adenosine deoxyribonucleotides ide novoi biosynthesis	0.04	0.03
KEGG Pancreatic cancer Main Pathway	0.06	0.02
biocarta lissencephaly gene lis1 in neuronal migration and development Main Pathway	0.08	0.02
HGF Pathway Regulation of Cytoskeleton Cell Polarity and Cell Motility	0.04	0.02
KEGG Hedgehog signaling Main Pathway	0.01	−0.01
KEGG Circadian rhythm Main Pathway	−0.11	−0.06

**Table 7:** “Top-10” of the most and the least correlated molecular pathways between RNA and protein expression levels calculated for Uterine Corpus Endometrial Carcinoma biosamples.

Pathway	Pearson	Spearman
Tumor Infiltration Pathway	0.82	0.77
NCI Signaling events mediated by PTP1B Main Pathway	0.72	0.74
L-kynurenine degradation	0.68	0.74
reactome Interleukin receptor SHC signaling Main Pathway	0.72	0.73
NCI N cadherin signaling events Pathway (regulation of cell adhesion)	0.67	0.73
reactome Pyrimidine catabolism Main Pathway	0.70	0.73
reactome ISG15 antiviral mechanism Main Pathway	0.79	0.73
NCI Aurora A signaling Pathway (protein catabolic process)	0.67	0.72
FCGR3A-mediated phagocytosis	0.65	0.71
ATM Pathway G2-Mitosis progression	0.59	0.71
reactome RNA Polymerase II Promoter Escape Main Pathway	0.17	0.11
reactome Abortive elongation of HIV 1 transcript in the absence of Tat Main Pathway	0.14	0.11
biocarta role of erbb2 in signal transduction and oncology Main Pathway	0.12	0.10
reactome IRAK2 mediated activation of TAK1 complex Main Pathway	0.21	0.09
reactome IRAK2 mediated activation of TAK1 complex upon TLR7 8 or 9 stimulation Main Pathway	0.21	0.09
reactome RNA Pol II CTD phosphorylation and interaction with CE Main Pathway	0.18	0.09
reactome Synthesis secretion and inactivation of Glucagon like Peptide 1 GLP 1 Main Pathway	0.10	0.08
reactome Synthesis secretion and inactivation of Glucose dependent Insulinotropic Polypeptide GIP Main Pathway	0.10	0.08
reactome Dual incision reaction in TC NER Main Pathway	0.14	0.07
reactome Synthesis secretion and deacylation of Ghrelin Main Pathway	−0.06	−0.06

## Supplementary Materials

The following are available online at https://www.mdpi.com/article/10.3390/ijms23052611/s1.
