# Supplementary material for: Better Agreement of Human Transcriptomic and Proteomic Cancer Expression Data at the Molecular Pathway Activation Level
Source: Int J Mol Sci. 2022 Feb 26;23(5):2611. doi: 10.3390/ijms23052611 (PMC8910457; doi:10.3390/ijms23052611)
Supplement: Supplementary file 1 [file ijms-23-02611-s001.zip › ijms-1577422-supplementary.pdf]

Supplementary Materials for

Better agreement of human transcriptomic and proteomic cancer expression data at the molecular pathway activation level

Mikhail Raevskiy[1,2,3], Maxim Sorokin[2,3], Galina Zakharova[2], Victor Tkachev[4], Nicolas Borisov[5], Denis Kuzmin[5], Kristina Kremenchutckaya[5], Alexander Gudkov[2], Dmitry Kamashev[2,3], Anton Buzdin[2,3,5*]

Correspondence to: buzdin@oncobox.com

This PDF file includes:

Figures. S1 to S7

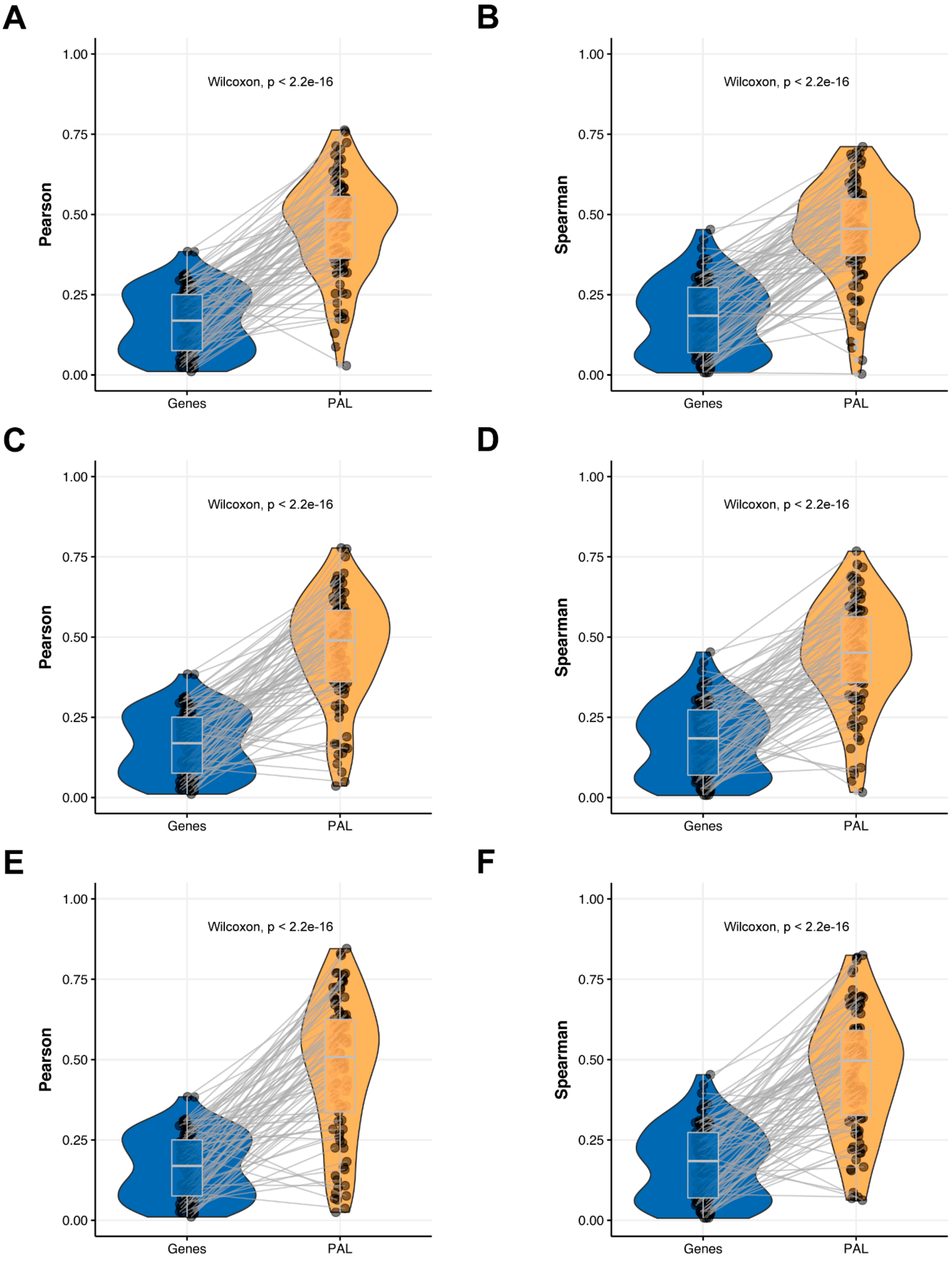

Figure S1. Paired gene-to-gene and PAL-to-PAL correlation between RNA and protein expression levels estimated within Breast Invasive Carcinoma biosamples using Pearson and Spearman correlation coefficients for a pool of molecular pathways having **(A-B)** 10, **(C-D)** 20, **(E-F)** 40 and more genes.

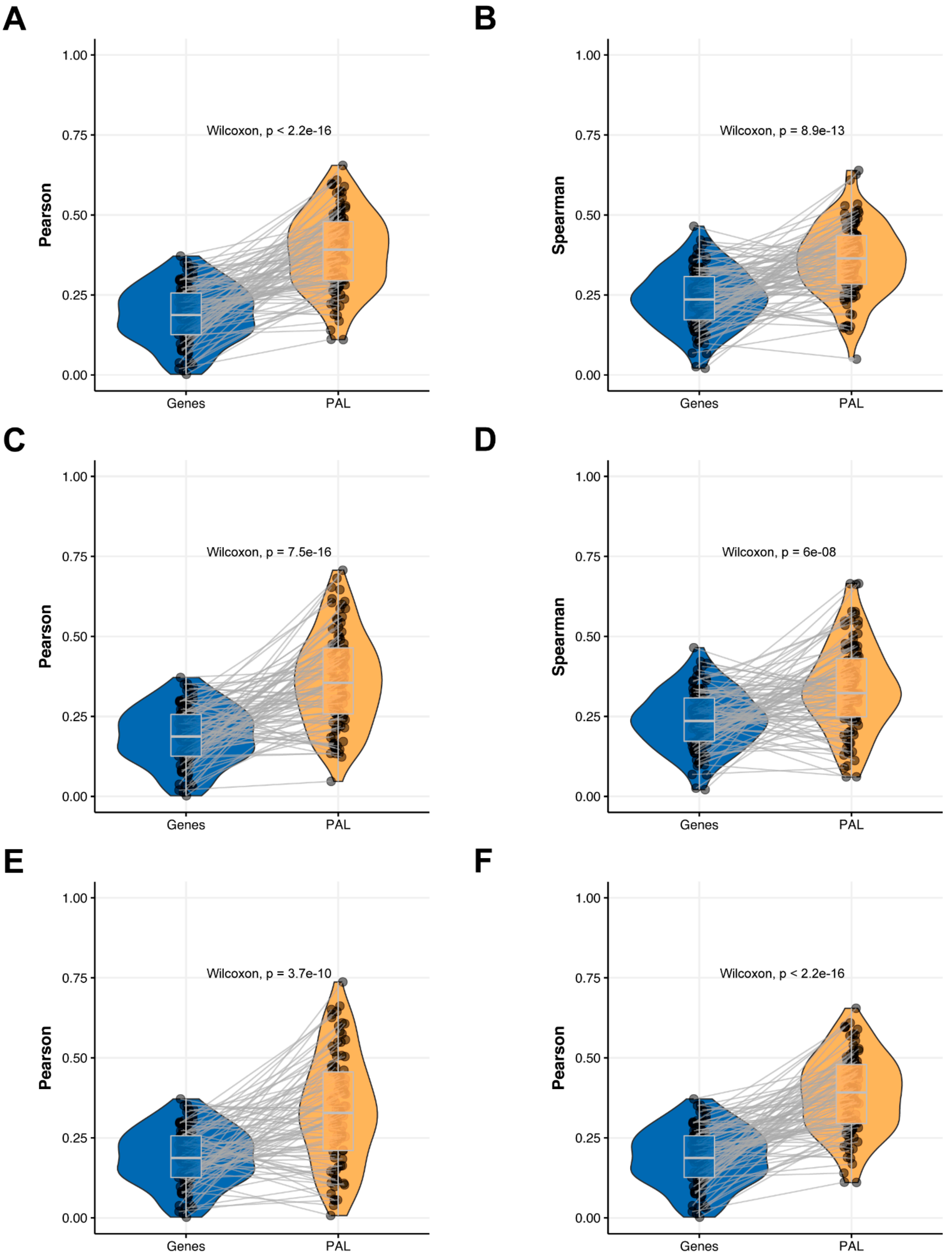

Figure S2. Paired gene-to-gene and PAL-to-PAL correlation between RNA and protein expression levels estimated within Glioblastoma Multiforme biosamples using Pearson and Spearman correlation coefficients for a pool of molecular pathways having **(A-B)** 10, **(C-D)** 20, **(E-F)** 40 and more genes.

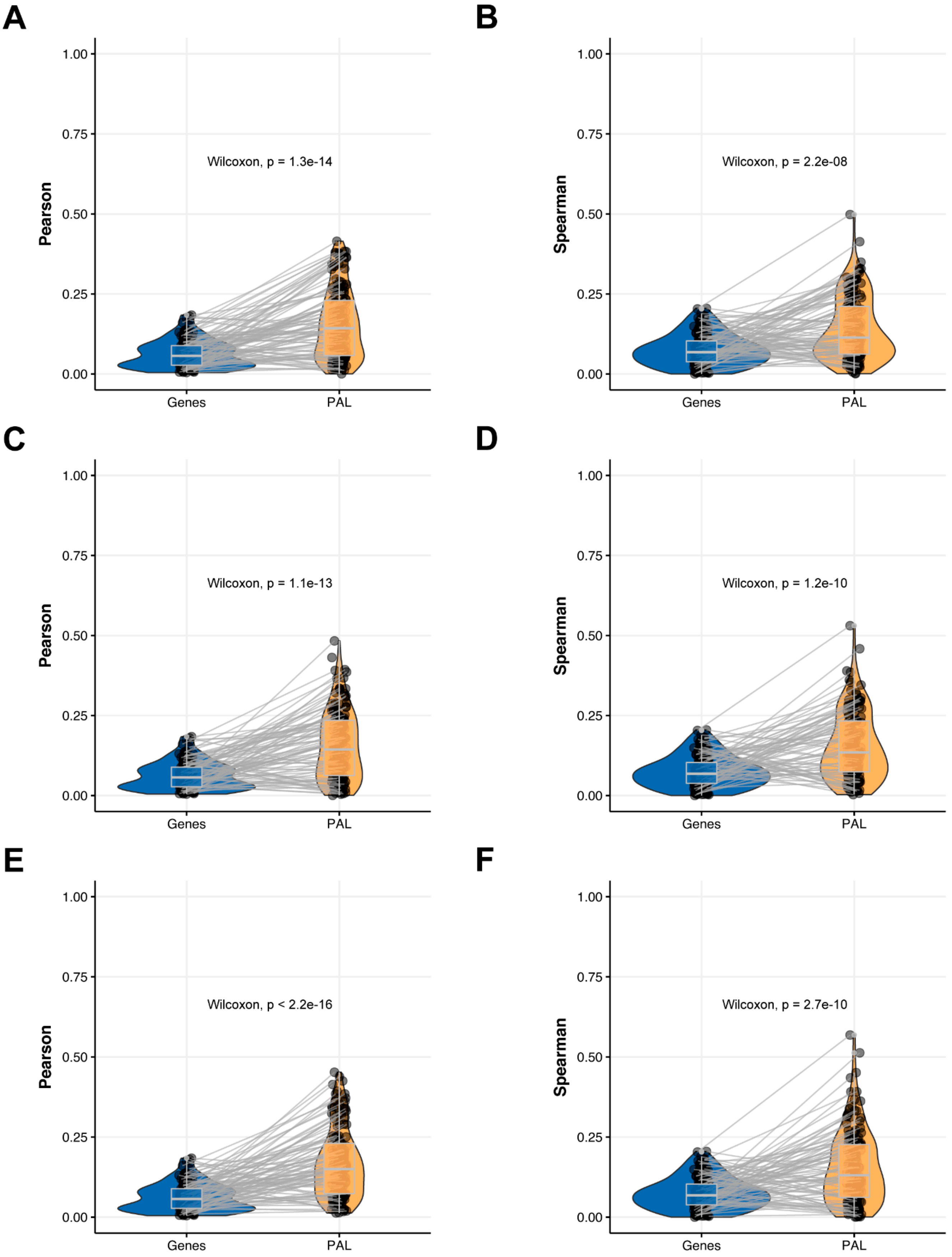

Figure S3. Paired gene-to-gene and PAL-to-PAL correlation between RNA and protein expression levels estimated within Hepatocellular Carcinoma biosamples using Pearson and Spearman correlation coefficients for a pool of molecular pathways having **(A-B)** 10, **(C-D)** 20, **(E-F)** 40 and more genes.

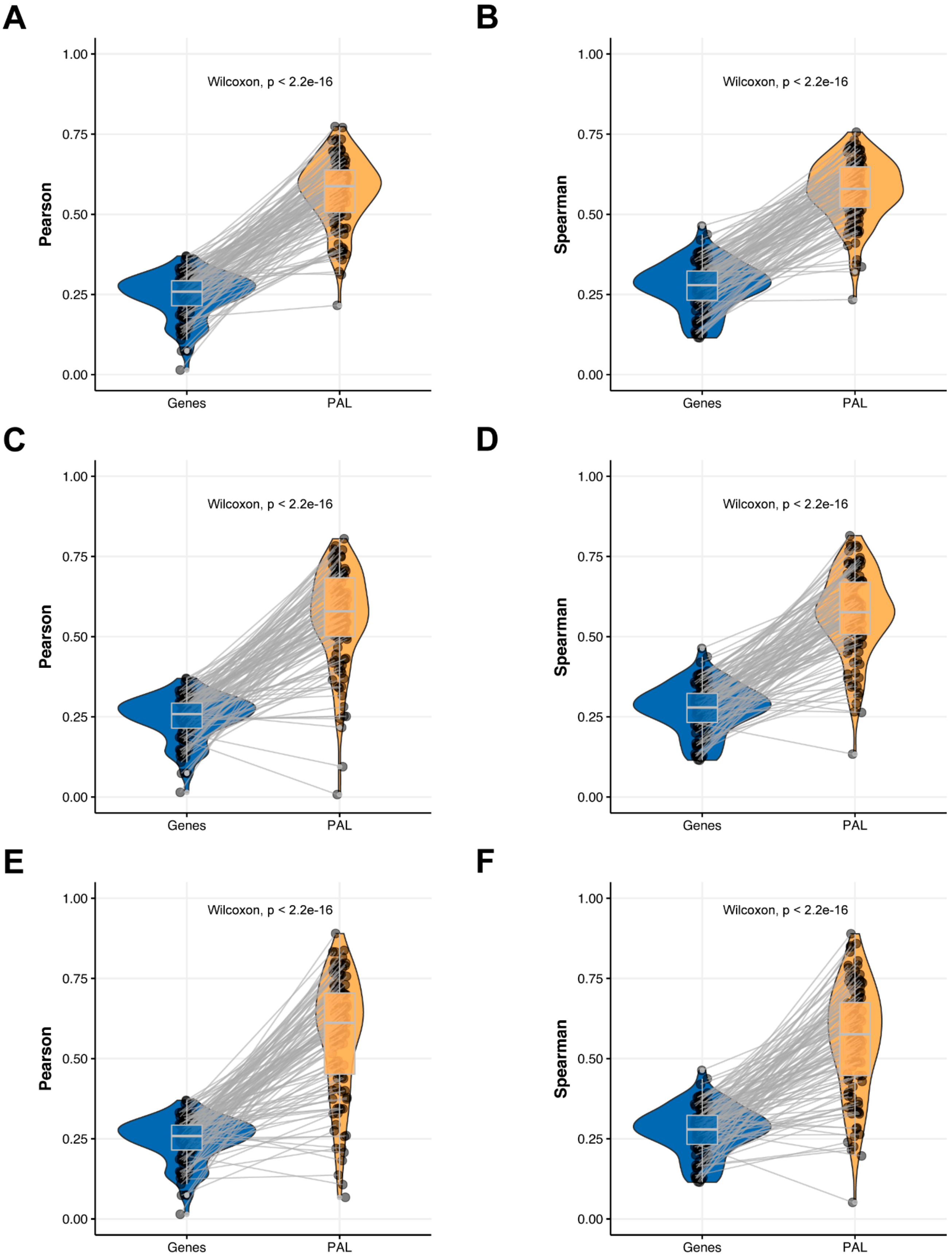

Figure S4. Paired gene-to-gene and PAL-to-PAL correlation between RNA and protein expression levels estimated within Lung Adenocarcinoma biosamples using Pearson and Spearman correlation coefficients for a pool of molecular pathways having **(A-B)** 10, **(C-D)** 20, **(E-F)** 40 and more genes.

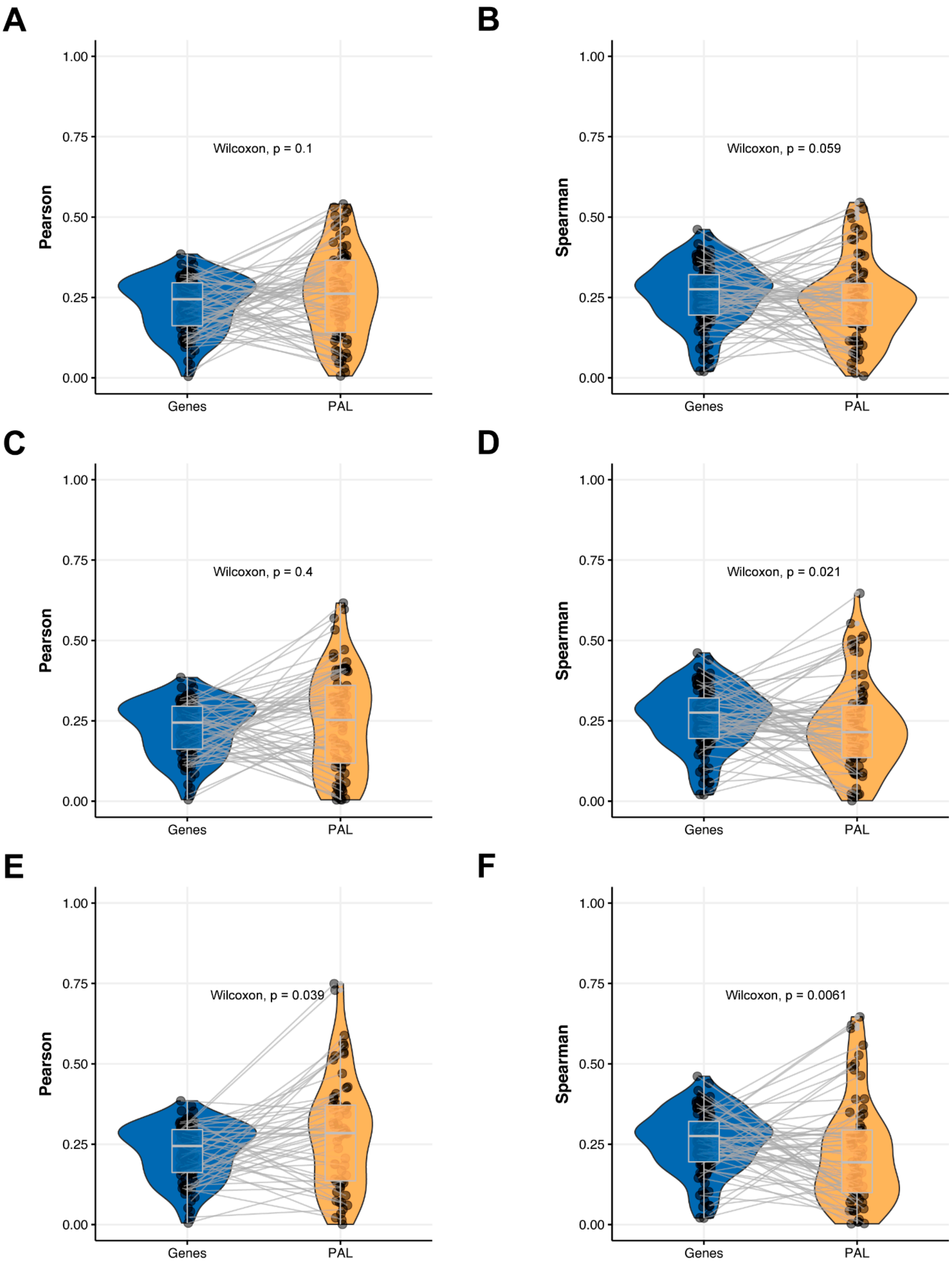

Figure S5. Paired gene-to-gene and PAL-to-PAL correlation between RNA and protein expression levels estimated within Ovarian Serous Cystadenocarcinoma biosamples using Pearson and Spearman correlation coefficients for a pool of molecular pathways having **(A-B)** 10, **(C-D)** 20, **(E-F)** 40 and more genes.

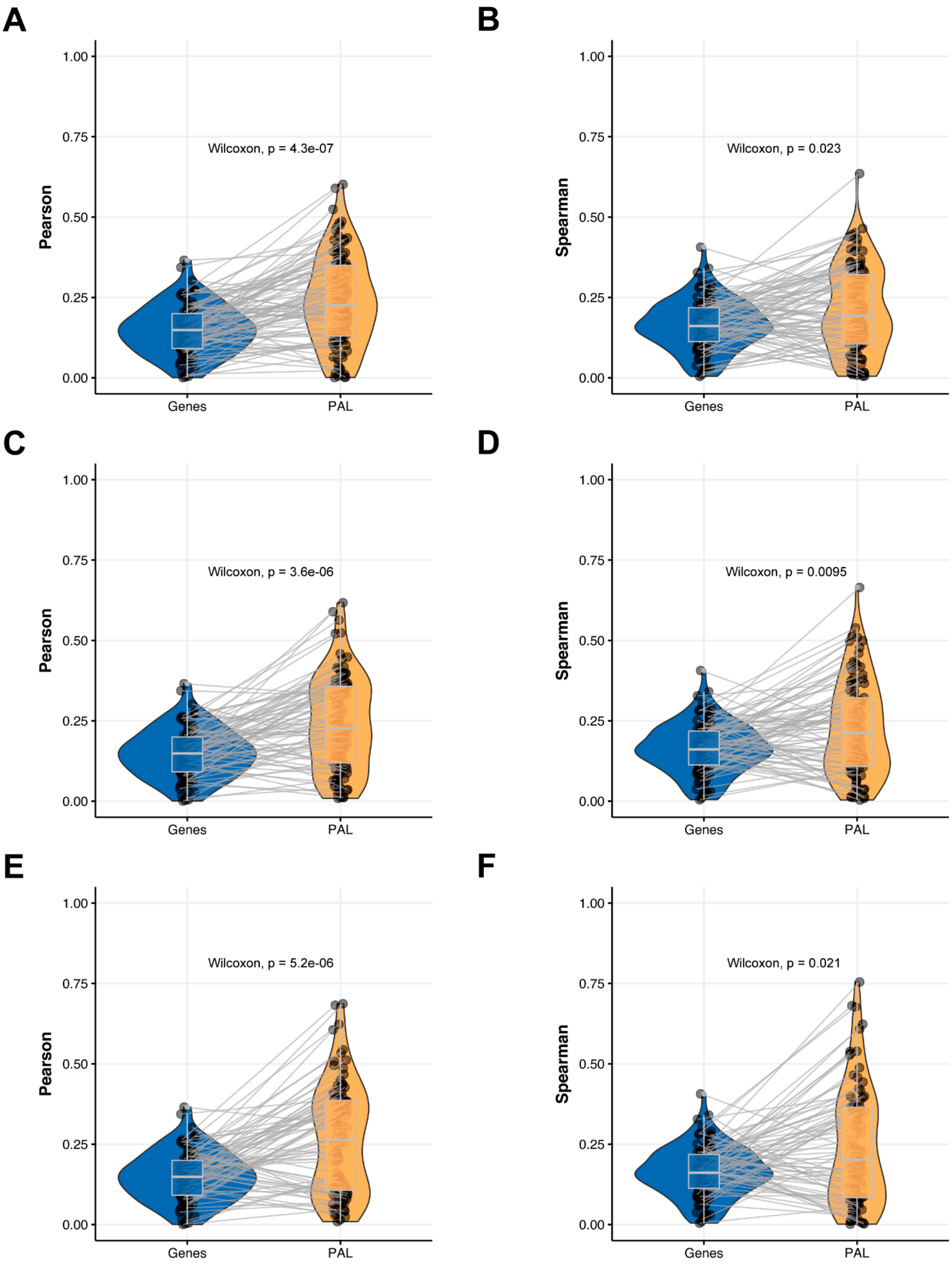

Figure S6. Paired gene-to-gene and PAL-to-PAL correlation between RNA and protein expression levels estimated within Pancreatic Ductal Adenocarcinoma biosamples using Pearson and Spearman correlation coefficients for a pool of molecular pathways having **(A-B)** 10, **(C-D)** 20, **(E-F)** 40 and more genes.

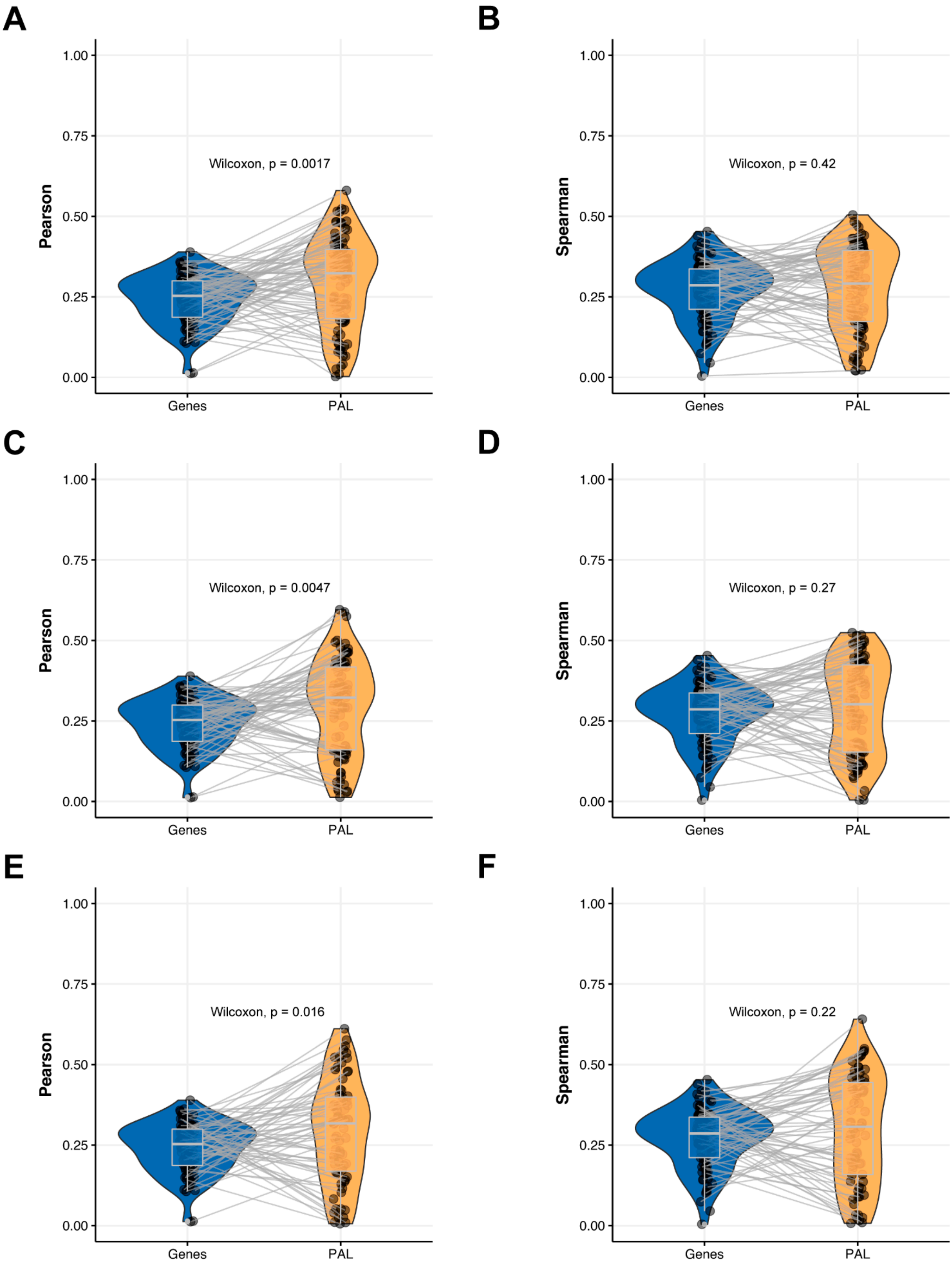

Figure S7. Paired gene-to-gene and PAL-to-PAL correlation between RNA and protein expression levels estimated within Uterine Corpus Endometrial Carcinoma biosamples using Pearson and Spearman correlation coefficients for a pool of molecular pathways having **(A-B)** 10, **(C-D)** 20, **(E-F)** 40 and more genes.
